# Neuronal Dysfunction Is Linked to the Famine-Associated Risk of Proliferative Retinopathy in Patients With Type 2 Diabetes

**DOI:** 10.3389/fnins.2022.858049

**Published:** 2022-05-05

**Authors:** Olena Fedotkina, Ruchi Jain, Rashmi B. Prasad, Andrea Luk, Marta García-Ramírez, Türküler Özgümüs, Liubov Cherviakova, Nadiya Khalimon, Tetiana Svietleisha, Tetiana Buldenko, Victor Kravchenko, Deepak Jain, Allan Vaag, Juliana Chan, Mykola D. Khalangot, Cristina Hernández, Peter M. Nilsson, Rafael Simo, Isabella Artner, Valeriya Lyssenko

**Affiliations:** ^1^Department of Clinical Science, Center for Diabetes Research, University of Bergen, Bergen, Norway; ^2^Department of Clinical Sciences, Lund University Diabetes Center, Skane University Hospital, Malmö, Sweden; ^3^Prince of Wales Hospital, Hong Kong Institute of Diabetes and Obesity, The Chinese University of Hong Kong, Hong Kong, Hong Kong SAR, China; ^4^Vall d’Hebron Research Institute and CIBERDEM, Barcelona, Spain; ^5^Chernihiv Regional Hospital, Chernihiv, Ukraine; ^6^City Hospital No. 2, Chernihiv, Ukraine; ^7^City Hospital No. 1, Chernihiv, Ukraine; ^8^Department of Health Care of Chernihiv Regional State Administration, Chernihiv, Ukraine; ^9^Komisarenko Institute of Endocrinology and Metabolism, Kyiv, Ukraine; ^10^Steno Diabetes Center Copenhagen, Copenhagen, Denmark; ^11^Shupyk National Healthcare University of Ukraine, Kyiv, Ukraine

**Keywords:** diabetic retinopathy, intrauterine exposure, famine, neuronal function, neurodegeneration

## Abstract

Persons with type 2 diabetes born in the regions of famine exposures have disproportionally elevated risk of vision-threatening proliferative diabetic retinopathy (PDR) in adulthood. However, the underlying mechanisms are not known. In the present study, we aimed to investigate the plausible molecular factors underlying progression to PDR. To study the association of genetic variants with PDR under the intrauterine famine exposure, we analyzed single nucleotide polymorphisms (SNPs) that were previously reported to be associated with type 2 diabetes, glucose, and pharmacogenetics. Analyses were performed in the population from northern Ukraine with a history of exposure to the Great Ukrainian Holodomor famine [the Diagnostic Optimization and Treatment of Diabetes and its Complications in the Chernihiv Region (DOLCE study), *n* = 3,583]. A validation of the top genetic findings was performed in the Hong Kong diabetes registry (HKDR, *n* = 730) with a history of famine as a consequence of the Japanese invasion during WWII. In DOLCE, the genetic risk for PDR was elevated for the variants in *ADRA2A*, *PCSK*9, and CYP2C19*2 loci, but reduced at *PROX1* locus. The association of *ADRA2A* loci with the risk of advanced diabetic retinopathy in famine-exposed group was further replicated in HKDR. The exposure of embryonic retinal cells to starvation for glucose, mimicking the perinatal exposure to famine, resulted in sustained increased expression of *Adra2a* and *Pcsk9*, but decreased *Prox1*. The exposure to starvation exhibited a lasting inhibitory effects on neurite outgrowth, as determined by neurite length. In conclusion, a consistent genetic findings on the famine-linked risk of *ADRA2A* with PDR indicate that the nerves may likely to be responsible for communicating the effects of perinatal exposure to famine on the elevated risk of advanced stages of diabetic retinopathy in adults. These results suggest the possibility of utilizing neuroprotective drugs for the prevention and treatment of PDR.

## Introduction

Patients with type 2 diabetes are at high risk of vision-threatening proliferative diabetic retinopathy (PDR) leading to blindness. The diabetic retinopathy (DR) remains a leading cause of the vision loss and preventable blindness in adults aged 20–74 years (Wong et al., 2016). The crude prevalence of the visual impairment and blindness caused by the DR has increased in recent years, mainly due to the increase of type 2 diabetes in low- and middle-income countries (Flaxman et al., 2017). Today, the laser therapy and anti-vascular endothelial growth factor (VEGF) intravitreous injections targeting neovascularization are the most commonly used treatments, despite that the recent clinical and research evidence advocate the important role of a tight cellular interaction between the different compartments of the neurovascular unit in the pathogenesis of PDR (Dodson, 2007; Simó et al., 2014; Simo et al., 2018). However, not all patients can afford and/or respond to these therapies and still effective preventive modalities are far to be optimal because the underlying causal mechanisms are not completely understood. Besides the modifying role of metabolic risk factors in diabetes, it is becoming evident that early life events such as intrauterine nutritional deprivation and stress exposures might contribute to the compromised programming of vasculature during fetal development and thereby increase susceptibility to the micro- and macro-vascular diseases in adults (Kistner et al., 2002; Mitchell et al., 2008; Gopinath et al., 2010). Thus, a perinatal exposure to famine was suggested to contribute to the rapid increase of type 2 diabetes prevalence in China—the current epicenter of the global diabetes epidemic (Zimmet et al., 2017). The Dutch hunger winter (1944–1945), the Chinese (1959–1961), and the Great Ukrainian (1932–1933) famine studies reported the link between the famine exposure at birth and the long-term adverse consequences in adults such as hyperglycemia, obesity, and dyslipidemia, but also cardiovascular disease and kidney dysfunction (Roseboom et al., 2001; Lumey et al., 2015; Zimmet et al., 2017). Our observations in individuals with type 2 diabetes, who experienced perinatal exposure to famine, demonstrated disproportionally elevated risk for PDR as compared to patients with type 2 diabetes that were born unexposed in two populations from Ukraine and Hong Kong (Fedotkina et al., 2021a).

In the present study, we investigated the possible underlying molecular mechanisms of the famine-associated risk of PDR by using genetic markers previously linked to glycemia or drug metabolism in the exposed to famine populations from Ukraine and Hong Kong. The changes in the expression of genes associated with PDR in the exposed to famine population were studied in the experimental primary cell culture model of embryonic retinal cells exposed to starvation for glucose and helped to confirm a molecular link between perinatal exposure to famine and PDR.

## Materials and Methods

### Study Populations

#### The Diabetes and Its Complications in the Chernihiv Region Study

The Diagnostic Optimization and Treatment of Diabetes and its Complications in the Chernihiv Region (DOLCE) is a population-based study of the patients with diabetes of all ages and their relatives in the Chernihiv region of northern Ukraine. Patients with diabetes and their first-degree relatives (parents, siblings, or children) were invited to participate. The recruitment started in November 2011 and ended in December 2014 (Fedotkina et al., 2021b). With the help of an endocrinologist and diabetes nurse, all participants completed a questionnaire covering their medical history, including the information on the family history of diabetes, anthropometric measurements (weight, height, and blood pressure), alcohol intake, smoking, medication for diabetes, hypertension, and hyperlipidemia. The information on co-morbidities was provided by primary care physicians, using the participants’ hospital discharge records as a primary source. Overall, 6,095 patients with diabetes (*n* = 785 type 1 diabetes, *n* = 4,297 type 2 diabetes, *n* = 62 unspecified, *n* = 951 healthy relatives) were enrolled in the study. The severity of DR was assessed using fundus photography. The PDR outcome was defined as proliferative retinopathy, or laser-treated DR, or blindness. The fasting samples were withdrawn for plasma glucose measurements and HbA1c; plasma and serum samples were stored at –80°C for further determinations of C-peptide, insulin, and lipid levels. All blood measurements were performed at the Department of Clinical Chemistry, Scania University Hospital Diabetes, Malmoe, Sweden (Laboratoriemedicin, 2020). Insulin resistance (HOMA2-IR) and beta-cell function (HOMA-B) were estimated based on C-peptide concentrations calculated using the HOMA calculator (Levy et al., 1998; HOMACalculator, 2021). Fasting blood-ethylenediaminetetraacetic acid (EDTA) samples were taken at the examination and were stored for DNA extraction from all patients with diabetes and their relatives. Genotyping was carried out at the Lund University Diabetes Center (LUDC), Department of Clinical Sciences, Diabetes and Endocrinology (Lund, Sweden). All participants provided a written informed consent. The study was approved by the local ethics committee (approval number for Ukraine: Dnr17/2011-09-14, for Norway: 2019/28968).

### The Hong Kong Diabetes Register: Validation Cohort for Genetic Findings

The Hong Kong Diabetes Registry (HKDR) was established in 1994 at the Diabetes and Endocrine Center, the Prince of Wales Hospital, Hong Kong Special Administrative Region (Luk et al., 2017; Fedotkina et al., 2021a). Patients with physician-diagnosed diabetes who attended the center for a comprehensive evaluation of diabetes complications were consecutively recruited. The referral sources included hospital- and community-based clinics. The detailed information including demographics, co-morbidities, and medication use was documented. The physical measurements, including vital signs and anthropometric parameters, were collected. The presence of diabetic retinopathy was examined by fundus photography and interpreted by trained endocrinologists. To keep the consistency with the previous report (Fedotkina et al., 2021a), advanced diabetic retinopathy was defined by fulfilling one or more of the following: Reduced visual acuity, PDR, pre-PDR, history of laser photocoagulation or presence of laser scar, and history of vitrectomy. Anti-VEGF treatment was not routinely used at the time that patients were recruited; hence it was not considered as advanced diabetic retinopathy. The fasting blood samples were obtained for plasma glucose, HbA1c, lipids, and renal function tests. A written informed consent was obtained from the patients at study enrollment. The HKDR was approved by the New Territories East Cluster Clinical Research Ethics Committee (reference number: 2007.339). The current dataset included 3,021 eligible participants from HKDR, 730 of them with available genetic information, and the selection of individuals was described in Supplementary Figure 1. The clinical characteristics of HKDR individuals are presented in Supplementary Table 1A. During the WWII period (1941–1945), Hong Kong experienced the famine exposure as a consequence of the Japanese invasion, which lasted for 3 years and 8 months.

#### Genetics

We performed gene-environment interaction to study the molecular susceptibility of PDR attributed to type 2 diabetes, which is more detrimental in the individuals at risk for type 2 diabetes after intrauterine undernutrition as a consequence of famine exposure (Fedotkina et al., 2021a). We analyzed a panel of 76 SNPs associated with type 2 diabetes, plasma glucose, and pharmacogenetics as a part of previously designed ANDIS panel of genetic loci reproducibly associated with risk of T2D in the 3,583 DOLCE participants (Ahlqvist et al., 2018). In DOLCE, the genotyping was performed using Mass Array iPLEX, and the Illumina Omni express array; genotyping of the individuals in HKDR was described elsewhere (van Zuydam et al., 2018). The schematic overview of genetic analyses flow in the DOLCE cohort was represented on the Supplementary Figure 2. Allele frequencies of SNPs used in the final analyses did not differ between groups that had or had not been perinatally exposed to famine, ruling out the contributing effects of at risk alleles to the excessive loss of susceptible individuals (*p* > 0.01) (Supplementary Table 2).

### *In vitro* Expression Measurements in Human Retinal Tissue

To rule out the effect of type 2 diabetes *per se* on expression of identified genes in the human retina in the patients without PDR, we obtained and analyzed postmortem retinal tissue from the Blood and Tissue Bank of Vall d’Hebron University Hospital, Barcelona, Spain. The procedure for eye cup donation and for the handling of this biologic material is rigorously regulated by the protocol of donations of the Blood and Tissue Bank of the Catalan Department of Health and was approved by the ethics committee of Vall d’Hebron University Hospital (PR-AG-4/2010). The methods for RNA extraction and reverse-transcription quantitative polymerase chain reaction (RT–qPCR) have been previously reported (Hernández et al., 2016). A total of 10 donors with diabetes and 5 donors without diabetes matched by age and sex were included in the study. One eye cup was harvested to separate neuroretina from retinal pigment epithelium, and samples of both tissues were immediately frozen with liquid nitrogen and stored at -80°C. The tissue samples derived from this eye cup were used for the studies of gene expression in the neuroretina. The time from death to eye enucleation was less than 4 h.

#### RNA Quality Assessment

The concentration and purity of RNAs were obtained by spectrophotometry in the NanoDrop instrument (Thermo Fisher Scientific), which specifically measure absorbance using small sample volumes. The quality of the samples was validated by the RIN assessment. *RT–PCR*. The PCRs were performed with the cDNA obtained with a High Capacity Kit (Applied Biosystems, Madrid, Spain) with random hexamer primers in a Thermal Cycler 2720 (Applied Biosystems, Madrid, Spain). Also, TaqMan Assays exon–exon boundary (Applied Biosystems, Madrid, Spain) were used for amplification of ADRA2A Hs01099503_s1; CYP2C19 Hs00426380_m1; PCSK9 Hs00545399_m1; PROX_m1; Hs00896293_m1 and β-Actin housekeeping gene assay (Hs01060665_g1) also purchased from Applied Biosystems (Applied Biosystems, Madrid, Spain). The PCR was performed in a 7.900 HT Thermal Cycler Sequence Detection System with 384-well optical plates (Applied Biosystems, Madrid, Spain). Then it was noticed that CYP2C19 Hs00426380_m1 and PCSK9 Hs00545399_m1 measurements (Applied Biosystems, Madrid, Spain) were higher than 35 CTs, and therefore they were not considered for the analysis. The rest of the measurements (<33 CTs) were obtained by calculating relative quantifications (R.Q.) using the Ct method.

#### *In vitro* Starvation Experiments of Embryonic Retinal Cells

##### Ethical Permits

The study was approved by the local ethics committee (2018–579, 2016/891), and the experiments were performed in compliance with the animals in research: reporting *in vivo* experiments (ARRIVE) guidelines (Kilkenny et al., 2010). Given that glucose is an essential nutrient needed for cell growth and proliferation, we designed *ex vivo* exposure experiments in embryonic retinal cells starved for glucose. The mice retinas were isolated from E18.5 embryos, and retinal cells were starved for glucose in a neurobasal medium for 6 h, followed by culturing in normal glucose medium for 6 days. We investigated the acute effects of starvation and analyzed expression of genes associated with DR using RT–qPCR and measured axonal length 1 day after the end of starvation. The measurements were repeated 6 days after the end of starvation to obtain information on the long-term effects of starvation exposure.

##### Isolation and Culture of Retinal Cells

First, C57BL/6J mice were purchased from Charles River. The retinas were isolated from E18.5 mouse embryos and digested with 0.05% trypsin (ready-made, Gibco) for 15 min at 37°C. The digestion was terminated by adding Dulbecco’s modified Eagle’s medium (Gibco) supplemented with 25-mM sodium bicarbonate (Gibco), 25-mM 4-(2-hydroxyethyl)-1-piperazineethanesulfonic acid (HEPES) (Gibco), 10% fetal bovine serum (v/v, Hyclone), and 1% penicillin and streptomycin solution (v/v, Gibco). The cell suspension was filtered through 70-μM filter and centrifuged at 1,300 rpm for 5 min, resuspended in medium, and centrifuged. This was repeated twice and the cells were plated on poly L-lysine coated plates at a density of 2.0 × 10^6^ cell/cm^2^. Second, on the next day, the cells were washed 2 times with phosphate-buffered saline (PBS) and starved for glucose in neurobasal medium supplemented with B27 supplement lacking insulin, with 0.06 g/L-glutamine, 1% penicillin–streptomycin (v/v, Gibco), and 11-mM HEPES for 6 h. Finally, the cells were further cultured for 6 days in the complete neurobasal medium.

##### The RNA Isolation and RT-qPCR

Total RNA was isolated using the miRNeasy micro kit (Qiagen). Reverse transcription was carried out using the RevertAid First Strand cDNA synthesis kit (Thermo Fischer Scientific), following the manufacturer’s instructions using 500-ng total RNA. Taqman RT–qPCR system was used for gene expression quantification (ThermoFischer Scientific). The gene expression data were normalized against the expression of hypoxanthine-guanine phosphoribosyltransferase (HPRT). The experiments were repeated for *n* = 4–5, each in duplicate. The data were shown as a mean expression with SEM and were analyzed with Student’s *t*-test.

##### Immunocytochemistry

Cells were fixed with 4% paraformaldehyde for 15 min at room temperature and permeabilized with 0.2% Triton X-100 in PBS for 10 min. The cells were then blocked for 1 h in 5% fetal bovine serum in PBS, and incubated with a primary antibody—rabbit anti-β III tubulin antibody (Covance; 1:1,000, Catalog No. PRB-435P-100), chicken anti-β III tubulin antibody (Abcam; 1:1,000, Catalog No. ab117716), rabbit anti-Pcsk9 antibody (Abcam; 1:100, Catalog No. ab31762), rabbit anti-Adra2a antibody (Sigma-Aldrich; 1:100, Catalog No. A271) and mouse anti-Nestin antibody (Chemicon; 1:500), overnight at 4°C. The next day, the cells were rinsed 3 times with PBS and incubated with a secondary antibody [donkey anti-rabbit IgG Alexa 488 (ThermoFischer Scientific); goat anti-rabbit IgG Alexa 568 (ThermoFischer Scientific) or donkey anti-rabbit IgG Cy5 (Jackson ImmunoResearch)] 1:250 for 1 h at the room temperature. The nuclei were stained with 2 μg/ml Hoechst 33342. The cells were imaged using Zeiss LSM 780 laser scanning microscope (LSM) using either 20 × or 40 × objective.

##### Image Analysis

Bright-field images of the isolated retinal cells on day 2 were acquired using an Olympus IX73 microscope. The fluorescent images on day 7 were acquired using the LSM (Zeiss LSM 780) using the 20 × objective and ZEN software (Zeiss). Four images of the retinal cultures were acquired per sample, and the neurite extensions were quantified using Fiji with simple neurite tracer plug-in (Longair et al., 2011; Schindelin et al., 2012). The representative bright-field images were also acquired using LSM (Zeiss LSM 780).

#### Global DNA Methylation

Genome-wide DNA methylation analyses were performed using DNA extracted from peripheral blood lymphocytes (Gentra Autopure, Qiagene) of the DOLCE study participants using the Illumina Infinium 450 Bead Chip with Infinium assay (Illumina iScan) and the standard Infinium HD assay methylation protocol guide (part number 15019519, Illumina). The DNA was quantified using picogreen (DNA assay kit 2000, Invitrogen, Tecan Infinitie); 1-μg DNA was bisulfite-treated using the EZ DNA Methylation™ kit (Zymo Research), following the manufacturer’s instructions. The modified DNA was hybridized with the Illumina 450 K beadchips and scanned using Illumina iScan, according to the manufacturer’s protocol. The samples were randomly distributed on the arrays. The Infinium HumanMethylation450 BeadChip contains 485 577 probes with 99% coverage of RefSeq genes with the capacity for 12 samples per chip (Bibikova et al., 2011).

The GenomeStudio methylation module software of the Genome studio Genome Browser (NCBI build 37) was used to calculate the raw methylation score for each DNA methylation site, represented as the methylation β-value. The β-values were calculated as follows: β = intensity of the methylated allele (*M*) ÷ (Intensity of the unmethylated allele (*U*) + intensity of the methylated allele [(*M*) + 100]). All samples passed the GenomeStudio quality control steps based on the built-in control probes for staining, hybridization, extension, and specificity, and displayed high-quality bisulfite conversion efficiency with an intensity signal above 4,000 (Bibikova et al., 2011). The probes detected at *p* > 0.01, less than three beads in at least 5% samples per probe, non-CpG probes, single nucleotide polymorphism (SNP)-related probes, multi-hit probes, and allosomal CpG probes were filtered out. Overall, DNA methylation data were obtained for 411,923 probes. The background correction and beta mixture quantile normalization (BMIQ) to normalize the type I and type II probes was implemented using ChAMP (Teschendorff et al., 2012). The singular value decomposition (SVD) method was used to assess batch effect, and ComBat was implemented to correct multiple batch effects (Johnson et al., 2007; Teschendorff et al., 2009). Since genome-wide methylation analysis was performed using DNA obtained from the whole blood, the methylation status could potentially reflect the combination of blood cell types. Then RefbaseEWAS was hence implemented to correct for changes in the distribution of white blood cells between different subpopulations using DNA methylation signatures in combination with a previously obtained external validation set consisting of signatures from purified leukocyte samples (Houseman et al., 2015). To reduce the heteroscedasticity for highly methylated or unmethylated sites, β-values were converted to *M*-values in the lumi package for further analysis calculated as *M* = log_2_[β/(1 - β)] (Du et al., 2008, 2010).

### Statistical Analysis

A flowchart for the quality control and preparation of the DOLCE dataset for the statistical analysis is represented in Supplementary Figure 3. All subsequent episodes of famine exposure in Ukraine were combined into decades of births before 1950 (exposed to famine) and after 1950 (unexposed) as previously described (Fedotkina et al., 2021b). To study the association of genetic variants and perinatal exposure to famine on the risk of PDR in adulthood, the interaction term between SNPs and famine exposure (year of birth before or after 1950) was fitted using generalized estimation equation using sex, age at visit, diabetes duration, and HbA1c as covariates, and corrected for family relationships (R-package “gee” version 4.13–19; defining families as clusters and correlation structure as exchangeable) (VanderWeele and Knol, 2014; Carey and Ripley, 2019). The association of the genetic variants and the risk of PDR in individuals that had been born perinatally exposed or unexposed to famine was also assessed using effect size heterogeneity Q-statistics and quantified using *I*^2^-value. Bonferroni correction was used to adjust for multiple testing in genetic association tests, *p* < 0.05 was considered statistically significant. The relationship between the risk variants and famine exposure and degree of methylation, quantified as *M*-value, was calculated using linear regression. The relationship between the risk variants and type 2 diabetes was analyzed using logistic regression using sex and age as covariates, *p*-values were adjusted for multiple testing correction using the false discovery rate method. All reported *p*-values are two-sided. All analyses were performed using R software, plink v. 1.07 (Purcell et al., 2007; R Core Team, 2013).

## Results

### Genetic Variants and the Risk of Proliferative Diabetic Retinopathy in Offspring of Individuals With Perinatal Exposure to Famine

#### Discovery of Genetic Variants in the Diabetes and Its Complications in the Chernihiv Region Cohort

To gain understanding into the putative biology of the previously reported link between exposure to famine and the risk of PDR (Fedotkina et al., 2021a), we analyzed a panel of type 2 diabetes susceptibility genetic loci in 3,583 patients with type 2 diabetes from the DOLCE study. Among these patients, 1,758 (30% men) individuals had been perinatally exposed to famine, and 1,825 (35% men) were born during modern times and had not been perinatally exposed to famine. Among them, 67 (3.8%) and 41 (2.2%) individuals had PDR, respectively. The clinical characteristics of the participants in the DOLCE cohort are shown in Supplementary Table 1B.

We found four SNPs to be significantly associated with PDR in offspring to famine-exposed individuals in the DOLCE study (Table 1). The risk loci linked to increased risk of PDR were glucose-rising genotypes of *ADRA2A* rs10885122 (OR_*exposedvs.unexposed*_, 95% CI, 3.67, 1.77–7.63 vs. 0.45, 0.28-0.71, *p*_*interaction*_ = 0.003); genotypes associated with pharmacogenetic response to statin therapy at *PCSK9* rs2479409 (2.27, 1.26–4.06 vs. 0.59, 0.37–0.94, *p*_*interaction*_ = 0.021); and genotypes associated with reduced function of drug-metabolizing gene CYP2C19*2 rs4244285 (2.87, 1.23–6.68 vs. 0.48, 0.23–0.99, *p*_*interaction*_ = 0.040). By contrast, alleles linked to elevated glucose levels at *PROX1* rs340874 showed a reduced risk of PDR (0.54, 0.32–0.89 vs. 1.57, 1.05–2.35, *p*_*interaction*_ = 0.045) (Table 1). However, only rs10885122 on *ADRA2A* remained significant after Bonferroni correction in heterogeneity analysis (*p*_*heterogeneity*_ = 6.0 × 10^–5^). To rule out the potential associations of genotypes with the poor glycemic control of diabetes, heterogeneity analyses were also adjusted for the HbA1c level, and the results did not change (Supplementary Table 3).

**TABLE 1:** Genetic variants and the risk of PDR for offspring to parents exposed and unexposed to famine in the DOLCE cohort.

Gene	SNP	Risk allele	RAF	Exposed to famine	Unexposed	Interaction	Effect size heterogeneity	
				OR (90% CI)	OR (90% CI)	*p*	*I* ^2^	Qep
*ADRA2A*	rs10885122	G	0.86	3.67 (1.77–7.63)	0.45 (0.28–0.71)	0.003	94	0.00006*
*PCSK9*	rs2479409	G	0.35	2.27 (1.26–4.06)	0.59 (0.37–0.94)	0.021	89	0.00313
*PROX1*	rs340874	C	0.50	0.54 (0.32–0.89)	1.57 (1.05–2.35)	0.045	87	0.00642
CYP2C19*2	rs4244285	A	0.13	2.87 (1.23–6.68)	0.48 (0.23–0.99)	0.040	86	0.00826

All subsequent episodes of exposure to famine were combined covering the period before 1950 (exposed to famine) and after 1950 (unexposed). The odds ratios are obtained from interaction analyses of risk variants (additive model) and famine exposure adjusted for sex, age, and diabetes duration. The influence of genetic variants on the risk of advanced diabetic retinopathy in individuals that had been born exposed or not exposed to famine was verified using odds-ratio heterogeneity Q-statistics and quantified using I^2^-value. RAF, risk allele frequency. *Significant after adjustment for the multiple testing using Bonferroni correction (p < 0.05).

#### Replication of Genetic Findings in the Hong Kong Diabetes Register Cohort

Replication of the top variants in the HKDR conferred significantly increased risk of *ADRA2A* rs10885122 for advanced diabetic retinopathy in persons with type 2 diabetes who were born during famine, while no risk was observed in unexposed individuals (OR, 95% CI, 3.13, 1.12–13.2, *p*_*famine*_ = 0.026 vs. 0.94, 0.18–17.4, *p*_*non* famine_ = 0.953) (Table 2).

**TABLE 2:** Genetic variants and the risk of ADVDR for offspring to parents exposed and unexposed to famine in the HKDR cohort.

Gene	SNP	Risk allele	RAF	Exposed to famine		Unexposed		Interaction
				OR (90% CI)	*p*	OR (90% CI)	*p*	*p*
*ADRA2A*	rs10885122	G	0.94	3.13 (1.12–13.2)	0.0265	0.94 (0.18–17.4)	0.953	0.368
*PCSK9*	rs2479409	G	0.68	0.91 (0.59–1.42)	0.674	1.48 (0.46–5.37)	0.522	0.455
*PROX1*	rs340874	C	0.41	0.78 (0.51–1.17)	0.236	1.26 (0.44–3.69)	0.661	0.401
CYP2C19*2	rs4244285	A	0.31	0.61 (0.37–0.96)	0.0325	0.51 (0.08–1.71)	0.308	0.814

The odds ratios are obtained from interaction analyses of risk variants (additive model) and famine exposure adjusted for sex, age, and diabetes duration. ADVDR, advanced diabetic retinopathy; RAF, risk allele frequency.

#### Gene Expression in Human Retina of Patients With and Without Diabetes

Notably, expression level of the *ADRA2A* and *PROX1* genes did not significantly differ in the retinas from non-famine type 2 diabetes patients with mild non-proliferative DR and non-diabetic donors (Blood and Tissue Bank of Vall d’Hebron University Hospital) (Supplementary Table 4), ruling out the impact of diabetes condition *per se* on expression level of identified genetic variants in the human retina. Together, these results point at presence of hyperglycemia independent molecular mechanisms driving progression to PDR in people who were exposed to famine-related insults at birth.

### Exposure of Embryonic Retinal Cells to Starvation and Gene Expression

#### Establishment and Evaluation of a Primary Cell Culture Model

**FIGURE 1:**
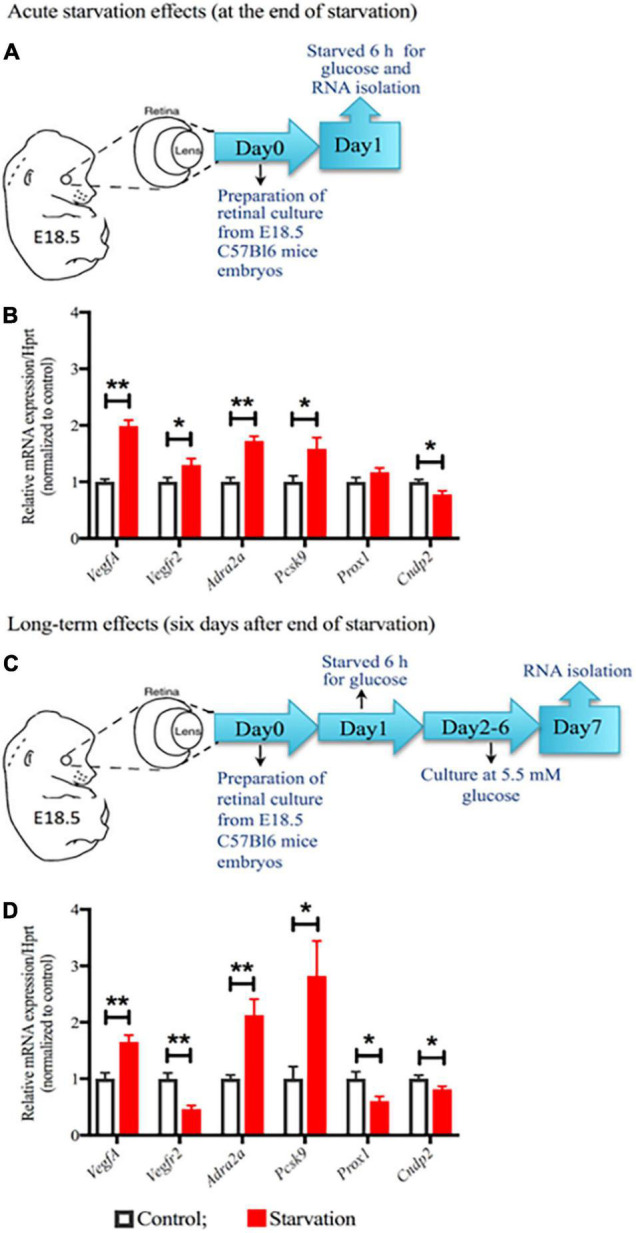
The impact of exposure to starvation for glucose on gene expression in embryonic retinal cells. **(A,C)** Schematic overview of the established and validated *in vitro* model of embryonic retinal cells exposed to glucose starvation to mimic the perinatal exposure to famine. Dissociated retinal cell cultures were prepared from E18.5 mouse embryos and plated on dishes coated with poly-L-lysine (day 0; as described in Supplementary Methods). The culture was starved for glucose for 6 h on day 1. RNA was isolated after the end of starvation on day 1 **(A)** or day 7 **(C)**. **(B,D)** Relative mRNA levels on day 1 **(B)** and day 7 **(D)**, determined by RT–qPCR (*n* = 5–6). Untreated cells were used as controls. **p < 0.01, *p < 0.05 (two-tailed paired Student’s *t*-test). All values are means + S.E.M.

The priming for future diseases during fetal development most likely includes modulation of gene expression. To study the potential mechanisms by which starvation could influence gene expression, we developed a primary cell culture model of starvation for glucose using mice embryonic retinal cells, since the human and mice retinal transcriptomes have shown remarkable similarity during development (Hoshino et al., 2017). As a proof-of-concept that our primary cell culture model mimics essential pathophysiological events implicated in the development of PDR (Aiello et al., 1994), we evaluated the expression of the pro-angiogenic genes *VegfA* and *Vegfr2* (Figure 1). These genes encode vascular endothelial growth factor A and its tyrosine kinase receptor, vascular endothelial growth factor 1R—the well-established pathogenic markers of PDR. We confirmed the upregulation of *VegfA* gene expression in response to glucose deprivation (Figure 1). Additionally, we analyzed expression of *Txnip*—a thioredoxin interaction protein ascribed a pathogenic role in diabetes and related complications, whose expression is strongly up-regulated by glucose in normal physiology (Parikh et al., 2007). As anticipated, *Txnip* expression was significantly reduced upon 6-h glucose deprivation (Supplementary Figure 4B). These observations confirmed that the primary cell culture model mimicking embryonic retinal cell starvation for glucose exhibit key pathogenic features (elevated VEGF) associated with PDR in humans.

#### Effects of Short Term (6-h) and Long-Term (6-Days) Starvation for Glucose on Gene Expression

In this model, short-term 6-h exposure to starvation for glucose resulted in the up-regulation of expression of *Adra2a* and *Pcsk9* (Figures 1A,B). Further, the expression of *Adra2a* and *Pcsk9* genes continued to be elevated in starved cells cultured in normal glucose medium for 6 days after the end of starvation, while the expression of the *Prox1* gene was significantly reduced in comparison with untreated (control) cells (Figures 1C,D).

#### Effect of Starvation for Glucose on Morphology of Neurons

**FIGURE 2:**
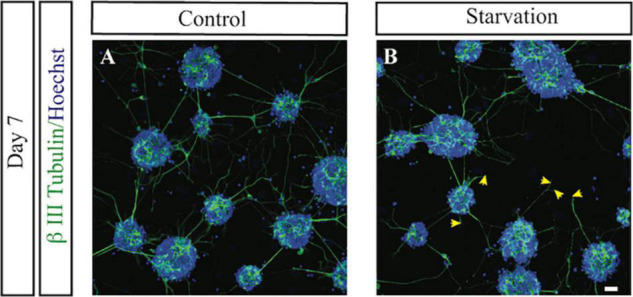
The impact of exposure to starvation for glucose on neurite outgrowth in embryonic retinal cells. Representative confocal LSM images of untreated (control) **(A)** and starved primary retinal cells **(B)** on day 7 of the experiment described in this figure, showing neurons (β III tubulin) and cell nuclei (Hoechst). The images are representative of 3–4 replicates. Scale bar, 20 μm. *n* = 3–4. Arrows indicate loss of neurite outgrowth.

**FIGURE 3:**
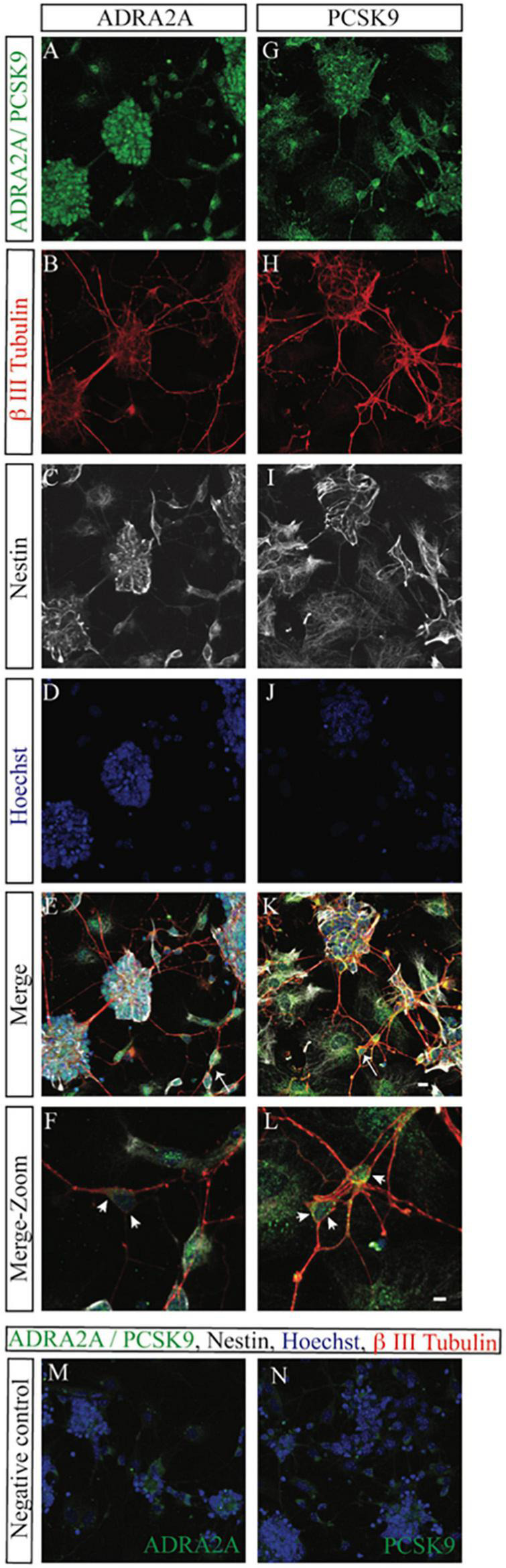
Immunocytochemistry of *ADRA2A* and *PCSK9* levels in control (untreated) primary mouse retinal cells on day 7. Representative confocal LSM images of primary mouse retinal cultures at day 7 showing expression of *ADRA2A* **(A)**, β III tubulin **(B)**, Nestin **(C)**, cell nuclei (Hoechst), **(D)** a merge image **(E)**, and a zoomed merge image **(F)**. **(G–L)** LSM images of primary mouse retinal cultures at day 7 showing expression of *PCSK9* **(G)**, β III tubulin **(H)**, Nestin **(I)**, cell nuclei (Hoechst) **(J)**, a merge image **(K)** and a zoomed merge image **(L)**. **(M,N)** Negative controls were treated without primary antibody and are shown as merge images. Scale bar = 10 μm or scale bar = 5 μm for zoomed images.

We also evaluated the effect of starvation on morphology of neurons using our primary cell culture model. We used the axonal length as a proxy for outgrowth capacity, serving as an indirect measurement of the ability of neurons to form extended connections—a hallmark of plasticity and adaptive processes in brain development (Prince, 1998). On average, the length of the longest neurite was 11-μm shorted 1 day after exposure to starvation, while the mean length of starved neurites was not significantly affected (Supplementary Figure 5). Long-term effects, nevertheless, revealed 3.7-μm shortening (*p* < 0.01) of the mean axonic length of starved retinal neurons (Supplementary Figure 5), suggesting sustained inhibitory effects of starvation on neurite outgrowth (Figure 2). Immunohistochemistry revealed that *ADRA2A* was expressed in both the neuron body and the axon, while the expression of *PCSK9* was higher at the plasma membrane of the neurons than at axon (Figure 3). Collectively, these observations suggested persistent effects of starvation exposure on the morphological properties of the neurons.

### Effects of Risk Variants on Methylation and Gene Expression

#### Methylation QTLs and Expression QTLs

A potential mechanism affecting changes in gene expression might involve epigenetic modifications of the DNA (Tobi et al., 2018). In a limited subset of DOLCE participants exposed and unexposed to famines (*n* = 51), we performed genome-wide methylation genotyping for exploratory analyses. Methylome analysis of DNA revealed differential methylation of the *ADRA2A* gene (*p* < 0.05) in famine-exposed compared with unexposed individuals (Supplementary Table 5A). To investigate the potential methylome QTL effects of identified SNPs, we performed look up in the publicly available mother-child ALSPAC database, which supported the association of methylome with genetic variants for all four genes (*ADRA2A*, *PCSK9*, CYP2C19*2, and *PROX1)* (Supplementary Table 6; Golding et al., 2001). Additionally, to investigate expression QTL effects, we performed look-up in the GTEX database, which deposited tissue biobank to study relationship between genetic variants and gene expression in diverse tissues in humans. These analyses showed effects of retinopathy-associated genotypes on increased gene expression for *PCSK9* (rs2479409) in skin fibroblasts, for CYP2C19*2 (rs4244285) in skin fibroblasts, liver, and stomach, and for *PROX1* (rs340874) in the brain (Supplementary Table 7; Consortium, 2015). The genotypes of *ADRA2A* or its proxies were not present in the GTEX database. Although expression of the gene in the retinal cells is not present in the GTEX database, observed eQTL effects emphasize the potential role of the identified genetic variants in the different organs and tissues, and thus plausible in the retina.

## Discussion

The results of this study highlight the importance of the neuronal dysfunction as a potential early event in the pathogenesis of microcirculatory abnormalities in adult type 2 diabetes patients with PDR whom, as children, were exposed to perinatal famine at birth. In this regard, the genetic and molecular immunohistochemistry findings of embryonic retinal cells support the idea that starvation for glucose insults during early retinogenesis might exhibit a lasting effect and act as triggers of subsequent diabetes-associated changes in the neurovascular unit in adults.

The findings of this study are in line with emerging evidence suggesting that neuroprotection could play a role in the treatment of early stages of DR, although current treatments for DR are mostly addressed to advanced disease (Simo et al., 2018, 2019). The central role for the neurovascular unit in the pathogenesis of DR has been previously discussed, but the exact pathogenic mechanisms are not entirely understood (Abcouwer and Gardner, 2014; Simo et al., 2014). The present study reveals a number of genes with neuronal and vascular functions that might influence risk of adult PDR, with *ADRA2A* being the strongest. The genetic association results from the two independent populations of Ukraine and Hong Kong exposed to famine at birth illuminated the most significant locus rs10885122 resided in the *ADRA2A* gene linked to function of nervous tissues. In the neuronal development of neonatal and adult hippocampus, α2ARA activity has been shown to regulate proliferation and survival of neural precursor cells (Jhaveri et al., 2014). These findings are in line with the reported neuroprotective effects of α2ARA agonist on Müller cells function after injury and also inhibition of apoptosis of the retinal ganglion cells (Nizari et al., 2016; Harun-Or-Rashid and Hallböök, 2018). This indicated the important role of neurons in the starvation-associated mechanisms linked to diabetic retinopathy and informed the ensuing experiments in the animal model. In this study, a significant and stable up-regulation of *Adra2a* expression was observed in the primary cell culture of embryonic retinal cells after starvation for glucose. Further, differential DNA methylation of *ADRA2A* upstream of SNP rs10885122 observed almost 70 years after perinatal exposure to famine could partly explain the long-lasting effects of famine on gene expression. It might be important to comment that we detected increased *ADRA2A* methylation in famine-exposed individuals, which could be referred as linked with transcriptional repression. However, there are plenty of examples of other forms of gene regulation as a function of CpG modulation, including transcriptional activation, which depends on the site and gene locus (Tirado-Magallanes et al., 2017). The results from this study support the previous observations and may pave the way for targeting α2ARA in the retina for the treatment of DR (Simo et al., 2019).

One possible mechanism by which early life exposure may exert programming effects on the risk of neuronal dysfunction later in life can involve impaired function of the stem cell progenitors (Dyer et al., 2003). In line with this, we found that the type 2 diabetes risk variant in the neuronal progenitor *PROX1* gene was associated with decreased risk of severe DR in offspring of individuals exposed to famine, while this variant increased the PDR risk in the unexposed group. The associations of *PROX1* variant showed same directionality of the effects in famine-exposed group from HKDR, even though did not reach statistical significance. Although our methylation studies might be underpowered to detect *PROX1* mQTL effects, in the publically available ALSPAC cohort, the risk variant in *PROX1* was associated with increased methylation as well as increased methylation of *PROX1* was seen in type 2 diabetes individuals as compared to healthy controls. Expression of *PROX1* is shown to be downregulated in islets from type 2 diabetes donors as compared to controls (Fadista et al., 2014). It is therefore tempting to speculate that methylation changes of *PROX1* gene during starvation could contribute to the increased gene expression, also acting as a protective mechanism to restore the pool of the stem cells.

One possibility to aid clinical management of PDR is that existing drugs will be beneficial for another disease. Interestingly, we found that variants of the *PCSK9* gene that affect lipid metabolism in the liver were associated with an increased risk of severe DR in individuals with famine exposure at birth (Zhang et al., 2016). Similarly, in the *ex vivo* model, the developmental changes induced by starvation exposure resulted in permanent up-regulation of *Pcsk9* expression in the retinal cells. A potential explanation could be that the *PCSK9* gene is methylated during starvation exposure and epigenetically regulated (Lohoff et al., 2017; Tobi et al., 2018). The manifestation of programmed fetal effects on adult phenotypes, nevertheless, may not be unmasked until later in life, after triggering by subsequent environmental factors (Gluckman et al., 2008). Therefore, this link is likely to be missed in most genetic studies conducted in populations of developed nations without subsequently experienced repeat exposure to famine. While *PCSK9* inhibitors have been introduced for the treatment of cardiovascular diseases, other potential effects of *PCSK9* inhibitors, such as DR treatment, should be explored.

It is important to note that the primary cell cultures were prepared from embryos at E18.5. At this stage, the retina constitutes not only retinal neurons but also Müller cells and endothelial cells (Blackshaw et al., 2004; Dakubo et al., 2008). In the present study, the immunostaining experiments displayed some isolated immunopositive profile of *ADRA2A*, *PCSK9* and *PROX 1* not colocalizing with beta3 tubulin. Notably, our recently published data on the global mRNA sequencing analyses from the same cellular model indicated that starvation for glucose caused marked transcriptomics changes in various retinal markers including vascular markers and allowed us to generate hypothesis of potential detrimental reprogramming of the entire neurovascular unit (Özgümüs et al., 2021).

**FIGURE 4:**
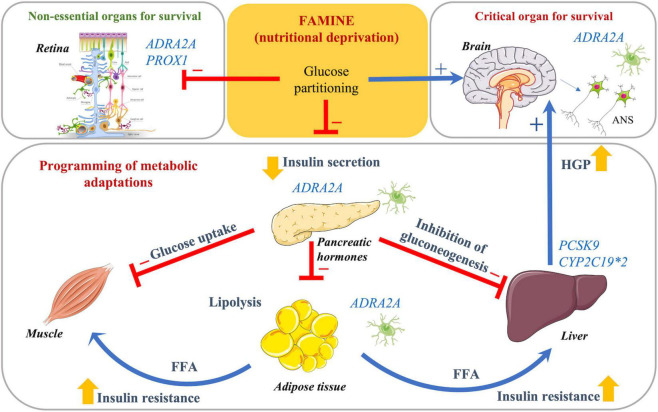
Schematic representation of the suggested mechanisms underlying association between perinatal famine exposure and the risk of proliferative retinopathy in adults with type 2 diabetes. An exposure to starvation induces a situation of nutritional deficits confronting the body to maintain metabolic balance by shunting the energy, i.e., glucose being the main source, from non-essential peripheral organs (including retina) to critical for survival organs (brain). Insulin secretion from the pancreas is reduced to slow down uptake of glucose in the periphery (muscle, liver, and adipose tissue), while endogenous hepatic glucose production (liver) is increased to partition and supply glucose to the brain. In addition to the adaptive insulin resistance, lipolysis is induced to release free fatty acids as backup system to be used as energy sources by non-essential organs and tissues; *ADRA2A* is abandonedly expressed in the neuronal tissues surrounding pancreatic islets, adipocytes, hepatocytes, and neuroretinal glial cells. This supports the concept of the metabolic re-programming of insulin secretion and action as well elevated lipolysis during early life as an adaptive mechanism to nutritional deprivation. The nerves may mediate these programming effects of increased predisposition to the risk of proliferative retinopathy in adults.

Several metabolic adaptations have been proposed to lie in the heart of fetal programming and developmental plasticity during intrauterine exposure to starvation (Figure 4). These are attributed to the situation when body is confronted with a nutritional challenge to maintain energy balance. The main mechanisms are centered around the goal of shunting energy resources derived using glucose from non-essential functions to the critical organs like brain, which accounts for greater than 80% of the body’s metabolism in the newborn (Tanner, 1978). Given that the insulin stimulates the glucose uptake by tissues throughout the body, the metabolic and signaling changes are required to modify insulin secretory capacity (de Rooij et al., 2006). To partition a glucose uptake between the brain and the insulin target organs (muscle, liver, and adipose tissue) mechanisms of inducing insulin resistance are taken place (Kuzawa, 2010). At the same time, the prolonged starvation leads to fat mobilization from the depots and releasing free fatty acids as backup system to be used as energy sources by these tissues (Kuzawa, 2010). Thereby, the neurons and the neuronal signaling network might play a central role in these energy partitioning mechanisms for developing brain as obligatory glucose user and nervous tissue as mediator of signals to the peripheral organs, e.g., lipolysis in the adipose tissue, insulin secretion from pancreatic beta cells, and inhibitory actions of insulin in the liver. In the study, the findings of genes involved in nervous tissues and lipid metabolism support the concepts of the metabolic re-programming of energy metabolism during early life as an adaptive mechanisms to nutritional deprivation, which may have lasting effects.

### Strengths and Limitations

Our genetic analyses were based only on selected first reports of established and reproducible genetic variants linked to risk of T2D, which represented only a fraction of type 2 diabetes susceptibility variants reported in the literature (Ahlqvist et al., 2018). Further genome wide association study (GWAS) analyses should support and unravel novel genetic loci contributing to the famine-associated risk of retinopathy. Although the methylation analyses supported the potential effects of epigenetic changes in the *ADRA2A* gene on the famine-associated risk of PDR, the subset with methylation data was underpowered to detect, particularly, small effects and therefore increased sample size would be required. Although the population of Hong Kong represents a unique validation cohort, the sample size with available genotyping was limited. Nevertheless, the association of *ADRA2A* locus with the advanced stages of diabetic retinopathy in famine-exposed individuals in the HKDR was similar to that observed in the DOLCE study reassuring the link between famine-related exposure and elevated risk of severity of diabetic retinopathy. Finally, the records on macular edema were not available in the DOLCE cohort; however, only seven patients with laser-treated retinopathy did not have information on PDR, which is less likely being able to substantially influence the results. In HKDR, patients with macular edema were excluded from the analyses.

The DOLCE participants with type 2 diabetes were from the Chernihiv region of northern Ukraine, a population that was affected by the Great Holodomor famine (Fedotkina et al., 2021a). The famine exposed group was defined based on the year of birth as we have previously demonstrated significantly elevated odds ratios for PDR in people from the exposed regions born before 1950 as opposed to those born after 1950 as compared to similar groups in the unexposed regions (Fedotkina et al., 2021a). These results indicated that other factors than age contributed to the elevated risks of PDR in the regions affected by the historical famine. Thus, one potential methodological caveat in both populational cohorts from Ukraine and Hong Kong could be that we did not have genetic information in a control group from the neighboring geographical regions of the same ethnicity not exposed to the famines. Additionally, there might be other cultural changes explaining differences between patients with diabetes in the DOLCE study born before and after 1950 such as socio-economical and behavioral factors. Thus, the stress hormones during the periods of famine insults in patients born before 1950 could *per se* induce epigenetic modifications (Ewald et al., 2014). Further, some therapeutics that became available to patients born after 1950 could potentially interact with the genetic susceptibility and thereby modify risks of PDR linked to *ADRA2A*, *PSCK9*, and CYP2C19*2 mutations or nurturing stemness effects of *PROX1* mutations. This enhances the importance of gene-environment and gene–drug interaction studies in discoveries of potential therapeutic mechanisms and drug targets.

In summary, we show the possible involvement of neuronal *ADRA2A*, *PCSK9*, and neuroprogenitor marker *PROX1* genes as molecular underpinnings in the pathogenesis of PDR. These findings may inform the development and testing of neuroprotective drugs relevant to the famine-exposed individuals or other individuals with progressive diabetes.

## Data Availability Statement

The datasets presented in this article are not readily available because of ethical considerations. Requests to access the datasets should be directed to corresponding author.

## Ethics Statement

The studies involving human participants were reviewed and approved by the Dnr17/2011-09-14—approval number for Ukraine (UNDR study), 2019/28968—approval number for Norway (UNDR study); reference number 2007.339 (HKDR study); PR-AG-4/2010 (expression measurements in human retinal tissue). The patients/participants provided their written informed consent to participate in this study. The animal study was reviewed and approved by the local ethics committee (2018-579, 2016/891).

## Author Contributions

OF did the statistical, genetic, and methylome analyses, data interpretation, and drafted the manuscript. RJ developed an experimental *in vitro* model and carried out statistical analyses, data interpretation, and drafted the manuscript. RP performed analyses of methylome data. MG-R, TÖ, CH, and RS carried out gene expression analyses. LC, NK, TS, and TB developed the study design, and collected the data. DJ assisted in immunohistochemistry analyses. VK, AV, and MK contributed to acquisition of the data. IA was responsible for the mice acquisition. PN participated in the study design and contributed to the editing of the manuscript. VL conceived and designed the study, planned the analyses, supervised all parts of the study, interpreted the data, and wrote the manuscript. All authors contributed to the interpretation of the data, and approved the final version of the manuscript.

## Conflict of Interest

The authors declare that the research was conducted in the absence of any commercial or financial relationships that could be construed as a potential conflict of interest.

## Publisher’s Note

All claims expressed in this article are solely those of the authors and do not necessarily represent those of their affiliated organizations, or those of the publisher, the editors and the reviewers. Any product that may be evaluated in this article, or claim that may be made by its manufacturer, is not guaranteed or endorsed by the publisher.

## References

[B1] Abcouwer S. F. Gardner T. W. (2014). Diabetic retinopathy: loss of neuroretinal adaptation to the diabetic metabolic environment. *Ann. N. Y. Acad. Sci.* 1311 174–190. 10.1111/nyas.12412 24673341 PMC4154702

[B2] Ahlqvist E. Storm P. Karajamaki A. Martinell M. Dorkhan M. Carlsson A. (2018). Novel subgroups of adult-onset diabetes and their association with outcomes: a data-driven cluster analysis of six variables. *Lancet Diabetes Endocrinol.* 6 361–369. 10.1016/S2213-8587(18)30051-2 29503172

[B3] Aiello L. P. Avery R. L. Arrigg P. G. Keyt B. A. Jampel H. D. Shah S. T. (1994). Vascular endothelial growth factor in ocular fluid of patients with diabetic retinopathy and other retinal disorders. *N. Engl. J. Med.* 331 1480–1487. 10.1056/NEJM199412013312203 7526212

[B4] Bibikova M. Barnes B. Tsan C. Ho V. Klotzle B. Le J. M. (2011). High density DNA methylation array with single CpG site resolution. *Genomics* 98 288–295. 10.1016/j.ygeno.2011.07.007 21839163

[B5] Blackshaw S. Harpavat S. Trimarchi J. Cai L. Huang H. Kuo W. P. (2004). Genomic analysis of mouse retinal development. *PLoS Biol.* 2:e247. 10.1371/journal.pbio.0020247 15226823 PMC439783

[B6] Carey V. J. Ripley B. (2019). *Generalized Estimation Equation Solver (Version 4.13-20) [R-package].* Available Online at: https://cran.r-project.org/web/packages/gee/gee.pdf (accessed October 26, 2020).

[B7] Consortium G. T. (2015). Human genomics. The Genotype-Tissue Expression (GTEx) pilot analysis: multitissue gene regulation in humans. *Science* 348 648–660. 10.1126/science.1262110 25954001 PMC4547484

[B8] Dakubo G. D. Mazerolle C. Furimsky M. Yu C. St-Jacques B. McMahon A. P. (2008). Indian hedgehog signaling from endothelial cells is required for sclera and retinal pigment epithelium development in the mouse eye. *Dev. Biol.* 320 242–255. 10.1016/j.ydbio.2008.05.528 18582859

[B9] de Rooij S. R. Painter R. C. Phillips D. I. Osmond C. Michels R. P. Godsland I. F. (2006). Impaired insulin secretion after prenatal exposure to the Dutch famine. *Diabetes Care* 29 1897–1901. 10.2337/dc06-0460 16873799

[B10] Dodson P. M. (2007). Diabetic retinopathy: treatment and prevention. *Diab. Vasc. Dis. Res.* 4 S9–S11. 10.3132/dvdr.2007.051 17935059

[B11] Du P. Kibbe W. A. Lin S. M. (2008). lumi: a pipeline for processing Illumina microarray. *Bioinformatics* 24 1547–1548. 10.1093/bioinformatics/btn224 18467348

[B12] Du P. Zhang X. Huang C.-C. Jafari N. Kibbe W. A. Hou L. (2010). Comparison of Beta-value and M-value methods for quantifying methylation levels by microarray analysis. *BMC Bioinformatics* 11:587. 10.1186/1471-2105-11-587 21118553 PMC3012676

[B13] Dyer M. A. Livesey F. J. Cepko C. L. Oliver G. (2003). Prox1 function controls progenitor cell proliferation and horizontal cell genesis in the mammalian retina. *Nat. Genet.* 34 53–58. 10.1038/ng1144 12692551

[B14] Ewald E. R. Wand G. S. Seifuddin F. Yang X. Tamashiro K. L. Potash J. B. (2014). Alterations in DNA methylation of Fkbp5 as a determinant of blood-brain correlation of glucocorticoid exposure. *Psychoneuroendocrinology* 44 112–122. 10.1016/j.psyneuen.2014.03.003 24767625 PMC4047971

[B15] Fadista J. Vikman P. Laakso E. O. Mollet I. G. Esguerra J. L. Taneera J. (2014). Global genomic and transcriptomic analysis of human pancreatic islets reveals novel genes influencing glucose metabolism. *Proc. Natl. Acad. Sci. U.S.A.* 111 13924–13929. 10.1073/pnas.1402665111 25201977 PMC4183326

[B16] Fedotkina O. Luk A. Jain R. Prasad R. B. Shungin D. Simó-Servat O. (2021a). Perinatal famine is associated with excess risk of proliferative retinopathy in patients with type 2 diabetes. *Acta Ophthalmol.* 100 e539–e545. 10.1111/aos.14948 34169655

[B17] Fedotkina O. Sulaieva O. Ozgumus T. Cherviakova L. Khalimon N. Svietleisha T. (2021b). Novel reclassification of adult diabetes is useful to distinguish stages of beta-cell function linked to the risk of vascular complications: the DOLCE study from northern Ukraine. *Front. Genet.* 12:637945. 10.3389/fgene.2021.637945 34276762 PMC8283002

[B18] Flaxman S. R. Bourne R. R. A. Resnikoff S. Ackland P. Braithwaite T. Cicinelli M. V. (2017). Global causes of blindness and distance vision impairment 1990-2020: a systematic review and meta-analysis. *Lancet Glob. Health* 5 e1221–e1234. 10.1016/S2214-109X(17)30393-5 29032195

[B19] Gluckman P. D. Hanson M. A. Cooper C. Thornburg K. L. (2008). Effect of in utero and early-life conditions on adult health and disease. *N. Engl. J. Med.* 359 61–73. 10.1056/NEJMra0708473 18596274 PMC3923653

[B20] Golding J. Pembrey M. Jones R. Team A. S. (2001). ALSPAC–the Avon longitudinal study of parents and children. I. study methodology. *Paediatr. Perinat. Epidemiol.* 15 74–87. 10.1046/j.1365-3016.2001.00325.x 11237119

[B21] Gopinath B. Baur L. A. Wang J. J. Teber E. Liew G. Cheung N. (2010). Smaller birth size is associated with narrower retinal arterioles in early adolescence. *Microcirculation* 17 660–668. 10.1111/j.1549-8719.2010.00062.x 21044220

[B22] Harun-Or-Rashid M. Hallböök F. (2018). Alpha 2-adrenergic receptor agonist brimonidine stimulates ERK1/2 and AKT signaling via transactivation of EGF receptors in the human MIO-M1 Müller cell line. *Curr. Eye Res.* 44 34–45. 10.1080/02713683.2018.1516783 30198788

[B23] Hernández C. Bogdanov P. Corraliza L. García-Ramírez M. Solà-Adell C. Arranz J. A. (2016). Topical administration of GLP-1 receptor agonists prevents retinal neurodegeneration in experimental diabetes. *Diabetes* 65 172–187. 10.2337/db15-0443 26384381

[B24] HOMACalculator (2021). *HOMA Calculator.* Available Online at: https://www.dtu.ox.ac.uk/homacalculator/ (accessed October 20, 2020).

[B25] Hoshino A. Ratnapriya R. Brooks M. J. Chaitankar V. Wilken M. S. Zhang C. (2017). Molecular anatomy of the developing human retina. *Dev. Cell* 43 763–779.e4. 10.1016/j.devcel.2017.10.029 29233477 PMC5776731

[B26] Houseman E. A. Kim S. Kelsey K. T. Wiencke J. K. (2015). DNA methylation in whole blood: uses and challenges. *Curr. Environ. Health Rep.* 2 145–154. 10.1007/s40572-015-0050-3 26231364

[B27] Jhaveri D. J. Nanavaty I. Prosper B. W. Marathe S. Husain B. F. Kernie S. G. (2014). Opposing effects of α2-and β-adrenergic receptor stimulation on quiescent neural precursor cell activity and adult hippocampal neurogenesis. *PLoS One* 9:e98736. 10.1371/journal.pone.0098736 24922313 PMC4055446

[B28] Johnson W. E. Li C. Rabinovic A. (2007). Adjusting batch effects in microarray expression data using empirical Bayes methods. *Biostatistics* 8 118–127. 10.1093/biostatistics/kxj037 16632515

[B29] Kilkenny C. Browne W. J. Cuthill I. C. Emerson M. Altman D. G. (2010). Improving bioscience research reporting: the ARRIVE guidelines for reporting animal research. *PLoS Biol.* 8:e1000412. 10.1371/journal.pbio.1000412 20613859 PMC2893951

[B30] Kistner A. Jacobson L. Jacobson S. H. Svensson E. Hellstrom A. (2002). Low gestational age associated with abnormal retinal vascularization and increased blood pressure in adult women. *Pediatr. Res.* 51 675–680. 10.1203/00006450-200206000-00003 12032260

[B31] Kuzawa C. W. (2010). “Beyond feast–famine: brain evolution, human life history, and the metabolic syndrome,” in *Human Evolutionary Biology*, ed. Muehlenbein M. P. (Cambridge: Cambridge University Press).

[B32] Laboratoriemedicin (2020). *Laboratoriemedicin.* Skåne: Scania Regional Council.

[B33] Levy J. C. Matthews D. R. Hermans M. P. (1998). Correct homeostasis model assessment (HOMA) evaluation uses the computer program. *Diabetes Care* 21 2191–2192. 10.2337/diacare.21.12.2191 9839117

[B34] Lohoff F. W. Sorcher J. L. Rosen A. D. Mauro K. L. Fanelli R. R. Momenan R. (2017). Methylomic profiling and replication implicates deregulation of PCSK9 in alcohol use disorder. *Mol. Psychiatry* 23 1900–1910. 10.1038/mp.2017.168 28848234 PMC5832488

[B35] Longair M. H. Baker D. A. Armstrong J. D. (2011). Simple Neurite Tracer: open source software for reconstruction, visualization and analysis of neuronal processes. *Bioinformatics* 27 2453–2454. 10.1093/bioinformatics/btr390 21727141

[B36] Luk A. O. Y. Lau E. S. H. Cheung K. K. T. Kong A. P. S. Ma R. C. W. Ozaki R. (2017). Glycaemia control and the risk of hospitalisation for infection in patients with type 2 diabetes: Hong Kong diabetes registry. *Diabetes Metab. Res. Rev.* 33:e2923. 10.1002/dmrr.2923 28731281

[B37] Lumey L. H. Khalangot M. D. Vaiserman A. M. (2015). Association between type 2 diabetes and prenatal exposure to the Ukraine famine of 1932-33: a retrospective cohort study. *Lancet Diabetes Endocrinol.* 3 787–794. 10.1016/S2213-8587(15)00279-X 26342852

[B38] Mitchell P. Liew G. Rochtchina E. Wang J. J. Robaei D. Cheung N. (2008). Evidence of arteriolar narrowing in low-birth-weight children. *Circulation* 118 518–524. 10.1161/circulationaha.107.747329 18625895

[B39] Nizari S. Guo L. Davis B. M. Normando E. M. Galvao J. Turner L. A. (2016). Non-amyloidogenic effects of α2 adrenergic agonists: implications for brimonidine-mediated neuroprotection. *Cell Death Dis.* 7:e2514. 10.1038/cddis.2016.397 27929541 PMC5260990

[B40] Özgümüs T. Sulaieva O. Jain R. Artner I. Lyssenko V. (2021). Starvation to glucose reprograms development of neurovascular unit in embryonic retinal cells. *Front. Cell Dev. Biol.* 9:726852. 10.3389/fcell.2021.726852 34869314 PMC8636675

[B41] Parikh H. Carlsson E. Chutkow W. A. Johansson L. E. Storgaard H. Poulsen P. (2007). TXNIP regulates peripheral glucose metabolism in humans. *PLoS Med.* 4:e158. 10.1371/journal.pmed.0040158 17472435 PMC1858708

[B42] Prince M. (1998). Is chronic low-level lead exposure in early life an etiologic factor in Alzheimer’s disease? *Epidemiology* 9 618–621. 10.1097/00001648-199811000-00009 9799170

[B43] Purcell S. Neale B. Todd-Brown K. Thomas L. Ferreira M. A. Bender D. (2007). PLINK: a tool set for whole-genome association and population-based linkage analyses. *Am. J. Hum. Genet.* 81 559–575. 10.1086/519795 17701901 PMC1950838

[B44] R Core Team (2013). *R: A Language and Environment for Statistical Computing.* Vienna: R Foundation for Statistical Computing.

[B45] Roseboom T. J. van der Meulen J. H. Ravelli A. C. Osmond C. Barker D. J. Bleker O. P. (2001). Effects of prenatal exposure to the Dutch famine on adult disease in later life: an overview. *Mol. Cell. Endocrinol.* 185 93–98. 10.1016/s0303-7207(01)00721-3 11738798

[B46] Schindelin J. Arganda-Carreras I. Frise E. Kaynig V. Longair M. Pietzsch T. (2012). Fiji: an open-source platform for biological-image analysis. *Nat. Methods* 9 676–682. 10.1038/nmeth.2019 22743772 PMC3855844

[B47] Simo R. Hernandez C. European Consortium for the Early Treatment of Diabetic Retinopathy (2014). Neurodegeneration in the diabetic eye: new insights and therapeutic perspectives. *Trends Endocrinol. Metab.* 25 23–33. 10.1016/j.tem.2013.09.005 24183659

[B48] Simo R. Hernandez C. Porta M. Bandello F. Grauslund J. Harding S. P. (2019). Effects of topically administered neuroprotective drugs in early stages of diabetic retinopathy: results of the EUROCONDOR clinical trial. *Diabetes* 68 457–463. 10.2337/db18-0682 30389750

[B49] Simo R. Stitt A. W. Gardner T. W. (2018). Neurodegeneration in diabetic retinopathy: does it really matter? *Diabetologia* 61 1902–1912. 10.1007/s00125-018-4692-1 30030554 PMC6096638

[B50] Simó R. Sundstrom J. M. Antonetti D. A. (2014). Ocular anti-VEGF therapy for diabetic retinopathy: the role of VEGF in the pathogenesis of diabetic retinopathy. *Diabetes Care* 37 893–899. 10.2337/dc13-2002 24652720

[B51] Tanner F. F. M. (1978). *Postnatal Growth Neurobiology.* Boston, MA: Springer.

[B52] Teschendorff A. E. Marabita F. Lechner M. Bartlett T. Tegner J. Gomez-Cabrero D. (2012). A beta-mixture quantile normalization method for correcting probe design bias in Illumina Infinium 450 k DNA methylation data. *Bioinformatics* 29 189–196. 10.1093/bioinformatics/bts680 23175756 PMC3546795

[B53] Teschendorff A. E. Menon U. Gentry-Maharaj A. Ramus S. J. Gayther S. A. Apostolidou S. (2009). An epigenetic signature in peripheral blood predicts active ovarian cancer. *PLoS One* 4:e8274. 10.1371/journal.pone.0008274 20019873 PMC2793425

[B54] Tirado-Magallanes R. Rebbani K. Lim R. Pradhan S. Benoukraf T. (2017). Whole genome DNA methylation: beyond genes silencing. *Oncotarget* 8 5629–5637. 10.18632/oncotarget.13562 27895318 PMC5354935

[B55] Tobi E. W. Slieker R. C. Luijk R. Dekkers K. F. Stein A. D. Xu K. M. (2018). DNA methylation as a mediator of the association between prenatal adversity and risk factors for metabolic disease in adulthood. *Sci. Adv.* 4:eaao4364. 10.1126/sciadv.aao4364 29399631 PMC5792223

[B56] van Zuydam N. R. Ahlqvist E. Sandholm N. Deshmukh H. Rayner N. W. Abdalla M. (2018). A genome-wide association study of diabetic kidney disease in subjects with type 2 diabetes. *Diabetes* 67 1414–1427. 10.2337/db17-0914 29703844 PMC6014557

[B57] VanderWeele T. J. Knol M. J. (2014). A tutorial on interaction. *Epidemiol. Methods* 3 33–72.

[B58] Wong T. Y. Cheung C. M. Larsen M. Sharma S. Simo R. (2016). Diabetic retinopathy. *Nat. Rev. Dis. Primers* 2:16012. 27159554 10.1038/nrdp.2016.12

[B59] Zhang L. Song K. Zhu M. Shi J. Zhang H. Xu L. (2016). Proprotein convertase subtilisin/kexin type 9 (PCSK9) in lipid metabolism, atherosclerosis and ischemic stroke. *Int. J. Neurosci.* 126 675–680. 10.3109/00207454.2015.1057636 26040332

[B60] Zimmet P. Z. El-Osta A. Shi Z. (2017). The diabetes epidemic in China is a public health emergency: the potential role of prenatal exposure. *J. Public Health Emerg.* 1:80. 10.21037/jphe.2017.10.01

