# Supplementary material for: Neuronal Dysfunction Is Linked to the Famine-Associated Risk of Proliferative Retinopathy in Patients With Type 2 Diabetes
Source: Front Neurosci. 2022 May 5;16:858049. doi: 10.3389/fnins.2022.858049 (PMC9119187; doi:10.3389/fnins.2022.858049)
Supplement: Supplementary file 1 [file Data_Sheet_1.pdf]

# 01 Supplementary Appendix

## Supplement to:

## Neuronal dysfunction is linked to the famine-associated risk of proliferative retinopathy in patients with type 2 diabetes

# Table of Contents

*Supplementary Figures* ........ *3*

**Supplementary Figure 1. Flowchart for quality control and preparation dataset for analysis in the Hong Kong Diabetes Register** ........ **3**

**Supplementary Figure 2. Schematic overview of genetic analyses flow to identify DNA variants influencing risk for diabetic retinopathy after perinatal exposure to famine.** ........ **4**

**Supplementary Figure 3. Flowchart for quality control and preparation dataset for analysis in DOLCE study** ........ **5**

**Supplementary Figure 4. Gene expression in the embryonic retinal cell cultures after the end of starvation exposure on day 1 or day 7.** ........ **6**

**Supplementary Figure 5. Neurite outgrowth in starved and control retinal cells on day 2 and day 7.** ........ **7**

**Supplementary tables** ........ **9**

**Supplementary Table 1A. Clinical characteristics of the participants in the Hong Kong Diabetes Register** ........ **9**

**Supplementary Table 1B. Clinical characteristics of the participants in the DOLCE study.** **10**

**Supplementary Table 2. Quality control and proxy SNPs used in the DOLCE study** ........ **11**

**Supplementary Table 3. DNA variants and the risk of proliferative diabetic retinopathy in individuals perinatally exposed or unexposed to famine.** ........ **18**

**Supplementary Table 4. Gene expression analyses in human retinal tissue.** ........ **21**

**Supplementary Table 5A. Association of DNA variants and famine exposure with the degree of methylation in the DOLCE study.** ........ **22**

**Supplementary Table 5B. Differentially methylated CpG sites in patients with type 2 diabetes and non-diabetic participants of the DOLCE study** ........ **23**

**Supplementary Table 6. Association of DNA variants with the degree of methylation in the Avon Longitudinal Study of Parents and Children cohort** ........ **25**

**Supplementary Table 7. Association of DNA variants with gene expression in the GTEX database.** ........ **27**

*References* ........ *28*

# Supplementary Figures

**Supplementary Figure 1. Flowchart for quality control and preparation dataset for analysis in the Hong Kong Diabetes Register.**

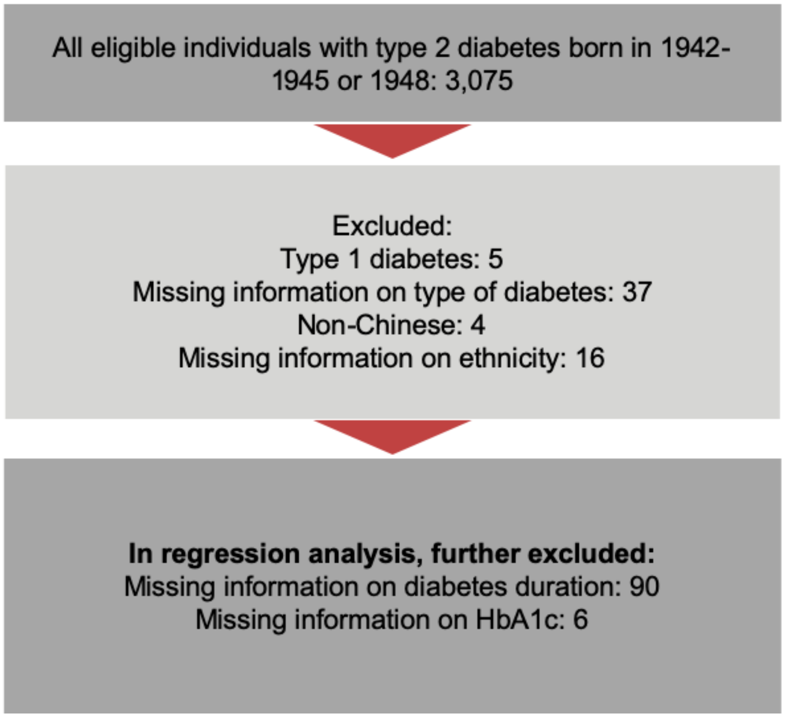

**Supplementary Figure 2. Schematic overview of genetic analyses flow to identify DNA variants influencing risk for diabetic retinopathy after perinatal exposure to famine.**

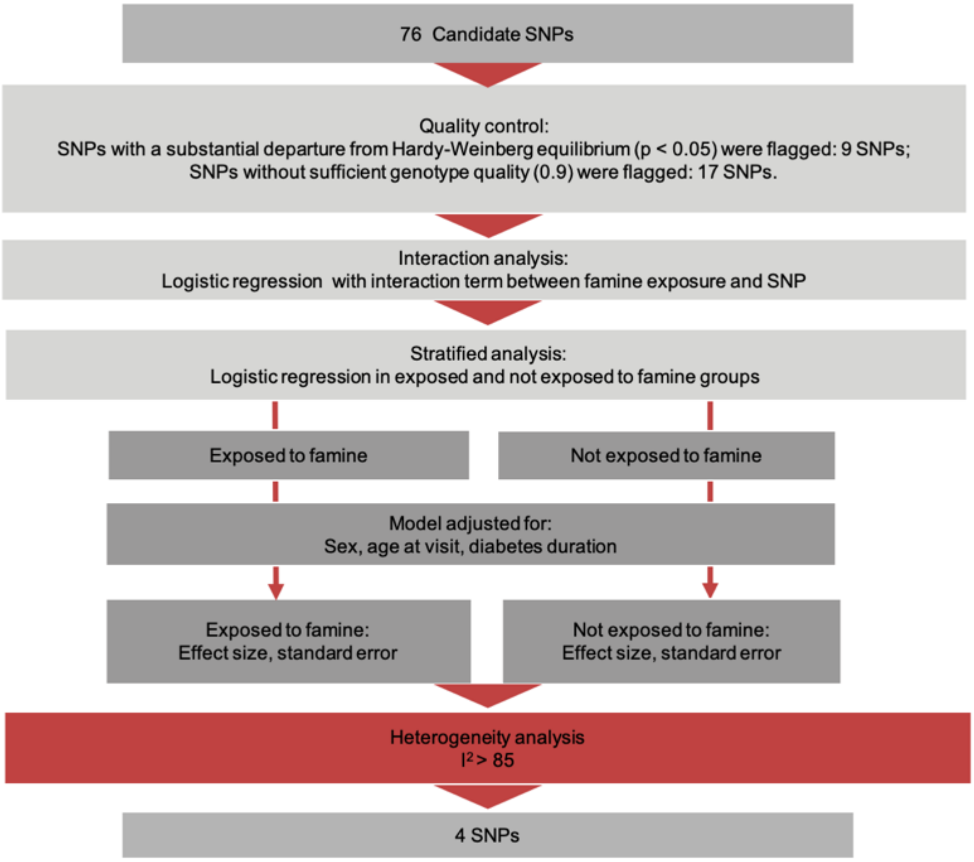

**Supplementary Figure 3. Flowchart for quality control and preparation dataset for analysis in DOLCE study.**

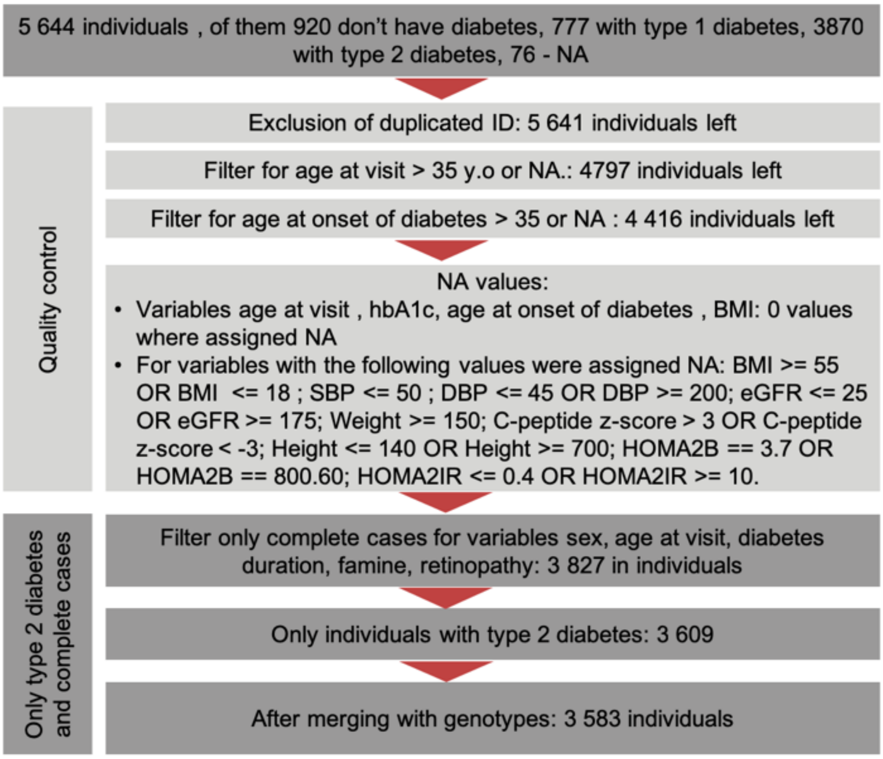

**Supplementary Figure 4. Gene expression in the embryonic retinal cell cultures after the end of starvation exposure on day 1 or day 7.**

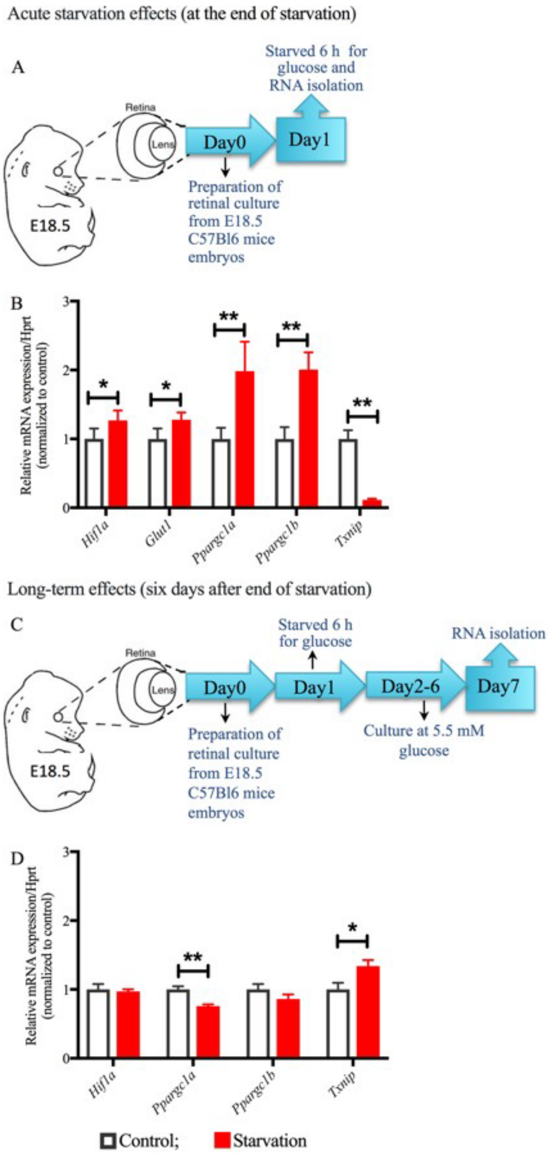


Gene expression in the embryonic retinal cell cultures after the end of starvation exposure on day 1 or day 7. (A,C)Schematic overview of the established and validated *in vitro* model of embryonic retinal cells exposed to glucose starvation to mimic the perinatal exposure to famine. Retinal cell cultures were prepared from E18.5 mouse embryos and plated on dishes coated on poly-l-lysine (day 0; as described in Supplementary Methods). The culture was starved for glucose for 6 h on day 1. RNA was isolated after the end of starvation on day 1 (A) or day 7 (C). (B,D) Relative mRNA levels on day 1 (B) and day 7 (D), determined by real-time RT-qPCR ($n$=5–6). Untreated cells were used as controls. **$P$<0·01, *$P$<0·05 (two-tailed paired Student's $t$-test). All values are means + s.e.m.

Schematic overview of the established and validated *in vitro* model of embryonic retinal cells exposed to glucose starvation to mimic the perinatal exposure to famine. Retinal cell cultures were prepared from E18.5 mouse embryos and plated on dishes coated on poly-L-lysine (day 0; as described in Supplementary Methods). The culture was starved for glucose for 6 h on day 1. RNA was isolated after the end of starvation on day 1 (A) or day 7 (B). (C,D) Relative mRNA levels on day 1 (C) and day 7 (D), determined by real-time RT-QPCR ($n$=5–6). Untreated cells were used as controls. **$P$<0.01, *$P$<0.05 (two-tailed paired Student's $t$-test). All values are means + s.e.m.

**Supplementary Figure 5. Neurite outgrowth in starved and control retinal cells on day 2 and day 7.**

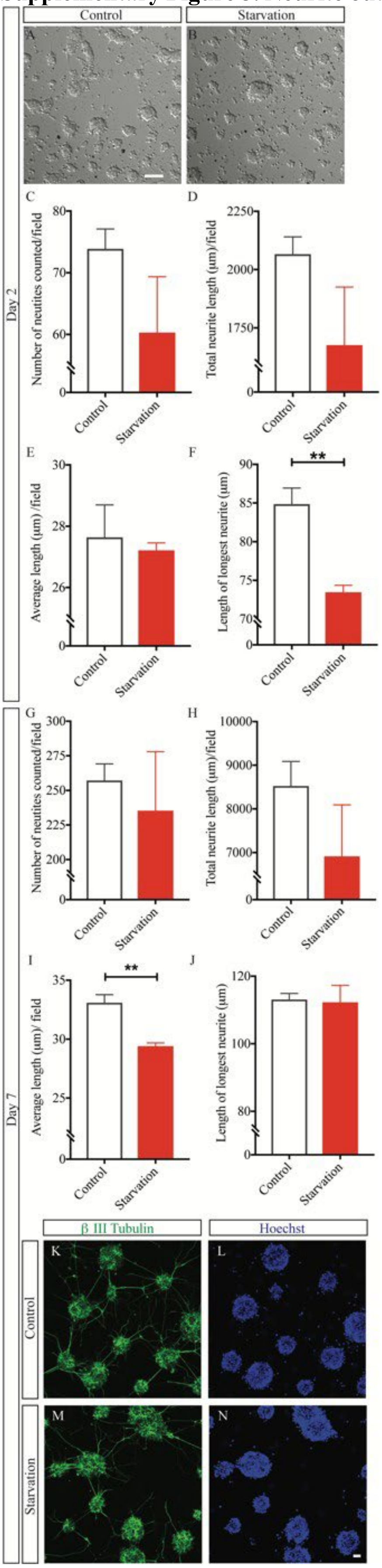


8

Representative confocal LSM images with 3-4 replicates on day 2 of *in vitro* retinal culture of experiment described in Figure 2, showing (A) untreated (control) and (B) starved primary mouse retinal cultures, isolated from E18.5 embryos. Scale bar, 100 µm. (C-F) Quantification at day 2 of *in vitro* retinal cultures showing (C) total number of neurites counted per field, (D) total neurite length counted (mm) per field, (E) average neurite length counted (mm) and (F) length of the longest neurite (mm) in control and starved cells. (G-J) Quantification of (G) total number of neurites counted per field, (H) total neurite length counted (mm) per field, (I) average neurite length counted (mm) and (J) length of the longest neurite (mm) in control and starved cells at day 7 of *in vitro* culture. (K-N) Representative confocal LSM images of control (K,L) and starved (M,N) primary mouse retinal cultures at day 7 showing expression of b III tubulin (K,M), cell nuclei (hoechst) (L,N). Scale bar, 20 µm. $n$ = 3-4. Untreated cells were used as controls. **$P < 0.01$, *$P < 0.05$ (two-tailed Student's $t$-test).

# Supplementary tables

| **Supplementary Table 1A. Clinical characteristics of the participants in the Hong Kong Diabetes Register.** | | | |
|---|---|---|---|
| **Phenotype** | **YOB** | | |
| | **All** | **1942 - 1945** | **1948** |
| N | 730 | 560 | 170 |
| Men, % | 49.7% (363) | 49.5% (277) | 50.6% (86) |
| ADVDR | 8.9% (65) | 10.2% (57) | 4.71% (8) |
| Age at visit | 56.2 ± 4.44 | 57.3 ± 4.05 | 52.7 ± 3.86 |
| Age at diagnosis | 48.2 ± 7.46 | 48.9 ± 7.34 | 45.7 ± 7.35 |
| Duration of diabetes | 7.89 ± 6.71 | 8.17 ± 6.84 | 6.96 ± 6.23 |
| BMI | 25.2 ± 3.86 | 25.2 ± 3.8 | 25.5 ± 4.03 |
| HEIGHT | 1.59 ± 0.0775 | 1.59 ± 0.0781 | 1.59 ± 0.0756 |
| HbA1C (%) | 7.7 ± 1.79 | 7.75 ± 1.77 | 7.52 ± 1.82 |
| HbA1C (mmol/mol) | 60.7 ± 19.5 | 61.3 ± 19.4 | 58.8 ± 19.9 |
| HDLC | 1.34 ± 0.361 | 1.34 ± 0.351 | 1.32 ± 0.392 |
| LDLC | 3.1 ± 1.1 | 3.11 ± 1.13 | 3.06 ± 0.967 |
| TG | 1.72 ± 1.64 | 1.76 ± 1.77 | 1.62 ± 1.12 |
| SBP | 136 ± 19.6 | 137 ± 20.1 | 135 ± 17.9 |
| DBP | 77.3 ± 11.1 | 76.8 ± 11.1 | 78.9 ± 10.8 |
| EGFR_CKDEPI | 82.6 ± 21.3 | 81.2 ± 21.7 | 87.2 ± 19.5 |

ADVDR – advanced diabetic retinopathy, BMI – body mass index, HbA1C - Hemoglobin A1C, HDLC - high-density lipoprotein cholesterol, LDLC - low-density lipoprotein cholesterol, TG - triglycerides, SBP - systolic blood pressure, EGFR_CKDEPI - estimated glomerular filtration rate, chronic kidney disease epidemiology collaboration.

| **Supplementary Table 1B. Clinical characteristics of the participants in the DOLCE study.** | | | |
|---|---|---|---|
| **Phenotype** | **All individuals** | **Exposed to famine** | **Unexposed** |
| Number of people (M, %) | 3,583 (32%) | 1,758 (30%) | 1,825 (35%) |
| Retinopathy (%) | 108 (3.01%) | 67 (3.8%) | 41 (2.2%) |
| Age at baseline (years) | 61.5 (9.1) | 68.9 (5.4) | 54.5 (5.9) |
| Age of diabetes (years) | 55 (9.3) | 60.5 (8.6) | 49.6 (6.5) |
| Duration of T2D (years) | 6.8 (6.5) | 8.6 (7.6) | 5.1 (4.7) |
| BMI (kg/m$^2$) | 31.3 (5.3) | 30.8 (4.9) | 31.9 (5.7) |
| Height (m) | 166 (8.3) | 164.7 (7.8) | 167.3 (8.6) |
| HbA1c (%) | 8.59 (2.39) | 8.54 (2.59) | 8.64 (2.21) |
| HbA1c (mmol/mol) | 70.44 (26.18) | 69.87 (28.29) | 70.93 (24.2) |
| HDL-cholesterol (mmol/l) | 1.26 (0.36) | 1.29 (0.36) | 1.21 (0.36) |
| LDL-cholesterol (mmol/l) | 3.92 (1.16) | 4.01 (1.2) | 3.81 (1.11) |
| Triglycerides (mmol/l) | 2.14 (1.57) | 1.99 (1.15) | 2.3 (1.91) |
| C-peptide (mmol/l) | 0.98 (0.469) | 0.957 (0.449) | 1.002 (0.487) |
| SBP (mm Hg) | 141.8 (17) | 143.6 (16.1) | 140.1 (17.7) |
| DBP (mm Hg) | 86.4 (10.7) | 86.7 (10.6) | 86.1 (10.8) |
| eGFR (ml/min/1.73m$^2$) | 74.992 (17.49) | 71.643 (15.618) | 78.371 (18.599) |
| HOMA2-B | 88.7 (54.9) | 87.3 (55.1) | 90.2 (54.7) |
| HOMA2-IR | 2.78 (1.32) | 2.68 (1.29) | 2.88 (1.34) |
| HOMA2-S | 45.5 (26.8) | 46.6 (26.2) | 44.4 (27.3) |

All episodes of famine All episodes of famine exposure were combined into decades of births before 1950 (exposed to famine) and after 1950 (unexposed).

**Supplementary Table 2. Quality control and proxy SNPs used in the DOLCE study.**

| Gene | SNP | Proxy SNP | Risk Allele | Alternative Allele | Genotype counts (11/12/22) | HW p-value | N | Call rate | RAF | RAF Famine exposed | RAF Unexposed | p-value | Gene description | Trait |
|---|---|---|---|---|---|---|---|---|---|---|---|---|---|---|
| *WFS1* | rs10010131 | - | G | A | 1894/2420/722 | 0.26 | 5036 | 0.92 | 0.62 | 0.63 | 0.62 | 0.42 | Wolfram syndrome 1 (wolframin) | T2D [1] |
| *SUGP1* | rs10401969 | - | C | T | 32/703/4283 | 0.55 | 5018 | 0.92 | 0.08 | 0.07 | 0.08 | 0.26 | SURP and G patch domain containing 1 | T2D [2] |
| *NAT2*6* | rs1041983 | rs6989506 | C | T | 1596/1487/347 | 1.00 | 3430 | 0.63 | 0.68 | 0.69 | 0.66 | 0.99 | N-acetyltransferase 2 (arylamine N-acetyltransferase) | Pharmacogenetic [3] |
| *GIPR* | rs10423928 | rs2287019 | A | T | 214/1242/1999 | 0.27 | 3455 | 0.63 | 0.24 | 0.25 | 0.24 | 0.55 | gastric inhibitory polypeptide receptor | 2hr glucose [4] |
| *CYP2C8*3* | rs10509681 | - | C | T | 40/591/2842 | 0.14 | 3473 | 0.64 | 0.1 | 0.1 | 0.1 | 0.49 | cytochrome P450, family 2, subfamily C, polypeptide 8 | Pharmacogenetic [5] |
| *CDKN2B* | rs10811661 | - | T | C | 3801/1128/95 | 0.29 | 5024 | 0.92 | 0.87 | 0.87 | 0.88 | 0.11 | cyclin-dependent kinase inhibitor 2B | T2D [6] |
| *MTNR1B* | rs10830963 | - | G | C | 585/2178/2312 | 0.04 | 5075 | 0.93 | 0.33 | 0.32 | 0.33 | 0.31 | melatonin receptor 1B | T2D [7] |
| *KLHDC5* | rs10842994 | - | C | T | 2848/1919/291 | 0.17 | 5058 | 0.93 | 0.75 | 0.76 | 0.76 | 0.38 | kelch domain containing 5 | T2D [2] |
| *ADRA2A* | rs10885122 | - | G | T | 3799/1180/83 | 0.46 | 5062 | 0.93 | 0.87 | 0.86 | 0.87 | 0.78 | α-2 adrenergic receptor | Fasting glucose [8] |
| *NOTCH2* | rs10923931 | rs835575 | T | G | 37/673/2559 | 0.39 | 3269 | 0.6 | 0.11 | 0.11 | 0.11 | 0.13 | notch 2 / ADAM metallopeptidase domain 30 | T2D [9, 10] |
| *CCND2* | rs11063069 | - | G | A | 267/1839/2885 | 0.26 | 4991 | 0.91 | 0.24 | 0.24 | 0.24 | 0.36 | Cyclin D2 | T2D [2] |

| | | | | | | | | | | | | | | |
|---|---|---|---|---|---|---|---|---|---|---|---|---|---|---|
| *FAM148B* | rs11071657 | - | A | G | 2152/2233/668 | 0.02 | 5053 | 0.92 | 0.65 | 0.65 | 0.64 | 0.46 | Solute carrier family 2 | Fasting glucose /T2D [8] |
| *HHEX/IDE* | rs1111875 | rs10882099 | C | T | 1375/1590/482 | 0.51 | 3447 | 0.63 | 0.63 | 0.62 | 0.65 | 0.06 | hematopoietically expressed homeobox | T2D [6] |
| *DCD* | rs1153188 | - | T | A | 328/1867/2876 | 0.29 | 5071 | 0.93 | 0.25 | 0.25 | 0.24 | 0.19 | dermcidin | T2D [9] |
| *CYP2C8*3* | rs11572080 | - | T | C | 17/432/2847 | 0.89 | 3296 | 0.6 | 0.07 | 0.07 | 0.08 | 0.29 | cytochrome P450, family 2, subfamily C, polypeptide 8 | Pharmacogenetic [5] |
| *CRY2* | rs11605924 | - | A | C | 1225/2521/1270 | 0.74 | 5016 | 0.92 | 0.5 | 0.5 | 0.48 | 0.26 | Cryptochrome-2 | Fasting glucose [8] |
| *ZFAND6* | rs11634397 | - | G | A | 2235/2232/603 | 0.21 | 5070 | 0.93 | 0.66 | 0.66 | 0.66 | 0.76 | zinc finger, AN1-type domain 6 | T2D [11] |
| *ADCY5* | rs11708067 | - | A | G | 3521/1353/127 | 0.88 | 5001 | 0.92 | 0.84 | 0.84 | 0.84 | 0.12 | Adenylate cyclase | T2D / 2hr glucose [4] |
| *SLC2A2* | rs11920090 | - | T | A | 3755/1168/94 | 0.76 | 5017 | 0.92 | 0.86 | 0.87 | 0.86 | 0.19 | solute carrier family 2 (facilitated glucose transporter), member 2 | Fasting glucose [8] |
| *ZMIZ1* | rs12571751 | - | A | G | 1387/2491/1137 | 0.78 | 5015 | 0.92 | 0.52 | 0.52 | 0.54 | 0.94 | zinc finger, MIZ-type containing 1 | T2D [2] |
| *MC4R* | rs12970134 | - | A | G | 284/1858/2936 | 0.67 | 5078 | 0.93 | 0.24 | 0.24 | 0.24 | 0.53 | melanocortin 4 receptor | T2D / BMI / waist circumference / insulin resistance [2, 12] |
| *SLC30A8* | rs13266634 | - | C | T | 2637/2057/356 | 0.10 | 5050 | 0.92 | 0.73 | 0.72 | 0.74 | 0.23 | solute carrier family 30 (zinc transporter), member 8 | T2D [13] |
| *TLE4* | rs13292136 | - | C | T | 4650/415/7 | 0.60 | 5072 | 0.93 | 0.96 | 0.96 | 0.96 | 0.55 | coiled-coil-helix-coiled-coil-helix | T2D [11] |

| | | | | | | | | | | | | | | |
|---|---|---|---|---|---|---|---|---|---|---|---|---|---|---|
| | | | | | | | | | | | | | domain containing 2 pseudogene 9 | |
| *GRB14* | rs13389219 | - | C | T | 1950/2353/742 | 0.46 | 5045 | 0.92 | 0.62 | 0.62 | 0.62 | 0.54 | growth factor receptor-bound protein 14 | T2D [2] |
| *HMGA2* | rs1531343 | - | C | G | 48/867/4157 | 0.68 | 5072 | 0.93 | 0.09 | 0.1 | 0.1 | 0.73 | high mobility group AT-hook 2 | T2D [11] |
| *CENTD2* | rs1552224 | - | A | C | 3737/1232/110 | 0.49 | 5079 | 0.93 | 0.86 | 0.86 | 0.85 | 0.23 | ArfGAP with RhoGAP domain, ankyrin repeat and PH domain 1 | T2D [11] |
| *KCNQ1* | rs163184 | rs163182 | G | T | 1276/2498/1253 | 0.67 | 5027 | 0.92 | 0.5 | 0.5 | 0.51 | 0.35 | potassium voltage-gated channel, KQT-like subfamily, member 1 | T2D [2, 14] |
| *DGKB* | rs17168486 | rs10276674 | T | C | 103/975/2364 | 0.86 | 3442 | 0.63 | 0.17 | 0.19 | 0.16 | 0.24 | diacylglycerol kinase, beta 90kDa | T2D [2] |
| *VPS13C* | rs17271305 | - | G | A | 748/2147/2024 | 0.00 | 4919 | 0.9 | 0.37 | 0.36 | 0.38 | 0.38 | vacuolar protein sorting 13 homolog C | 2hr glucose/2hrglucose [4] |
| *FADS1* | rs174550 | - | T | C | 2341/2228/509 | 0.54 | 5078 | 0.93 | 0.68 | 0.68 | 0.68 | 0.68 | Fatty acid desaturase | Fasting glucose /T2D [8] |
| *MC4R* | rs17782313 | - | C | T | 245/1711/2798 | 0.44 | 4754 | 0.87 | 0.23 | 0.23 | 0.23 | 0.98 | melanocortin 4 receptor | BMI / T2D [15, 16] |
| *NAT2*5* | rs1799929 | - | C | T | 1702/2477/893 | 0.89 | 5072 | 0.93 | 0.58 | 0.58 | 0.58 | 0.61 | N-acetyltransferase 2 (arylamine N-acetyltransferase) | Pharmacogenetic |
| *NAT2*6* | rs1799930 | - | A | G | 448/2100/2434 | 0.89 | 4982 | 0.91 | 0.3 | 0.3 | 0.3 | 0.30 | N-acetyltransferase 2 (arylamine N-acetyltransferase) | Pharmacogenetic [3] |

| | | | | | | | | | | | | | | |
|---|---|---|---|---|---|---|---|---|---|---|---|---|---|---|
| *PPARG* | rs1801282 | - | C | G | 3530/1289/140 | 0.10 | 4959 | 0.91 | 0.84 | 0.84 | 0.85 | 0.68 | peroxisome proliferator-activated receptor gamma | T2D [6] |
| *VEGF* | rs2010963 | - | G | C | 1919/1275/207 | 0.85 | 3401 | 0.62 | 0.75 | 0.75 | 0.75 | 0.94 | vascular endothelial growth factor A | DR [17] |
| *TMEM195* | rs2191349 | - | T | G | 1703/2460/879 | 0.86 | 5042 | 0.92 | 0.58 | 0.58 | 0.58 | 0.13 | Diacylglycerol kinase beta - TMEM195 | Fasting glucose [8] |
| *KCNQ1* | rs2237895 | - | A | C | 1629/2340/965 | 0.02 | 4934 | 0.9 | 0.57 | 0.58 | 0.56 | 0.34 | potassium voltage-gated channel, KQT-like subfamily, member 1 | T2D [14] |
| *KCNQ1* | rs231362 | - | G | A | 1371/2452/1035 | 0.33 | 4858 | 0.89 | 0.53 | 0.55 | 0.54 | 0.96 | potassium voltage-gated channel, KQT-like subfamily, member 1 | T2D [11] |
| *BCL11A* | rs243021 | - | A | G | 1117/2468/1414 | 0.53 | 4999 | 0.91 | 0.47 | 0.48 | 0.47 | 0.02 | B-cell CLL/lymphom a 11A (zinc finger protein) | T2D [11] |
| *BCL11A* | rs243088 | rs243083 | T | A | 1012/1725/732 | 0.97 | 3469 | 0.63 | 0.54 | 0.53 | 0.53 | 0.84 | B-cell CLL/lymphom a 11A | T2D [2] |
| *PCSK9* | rs2479409 | - | G | A | 626/2295/2137 | 0.81 | 5058 | 0.93 | 0.35 | 0.35 | 0.35 | 0.46 | proprotein convertase subtilisin/kexi n type 9 | LDL / TC [18] |
| *TLE1* | rs2796441 | - | G | A | 2051/2330/671 | 0.83 | 5052 | 0.92 | 0.64 | 0.64 | 0.64 | 0.48 | transducin-like enhancer of split 1 | T2D [2] |
| *IRS1* | rs2943641 | - | C | T | 2098/2290/683 | 0.14 | 5071 | 0.93 | 0.64 | 0.64 | 0.64 | 0.83 | insulin receptor substrate 1 | Fasting glucose/T2D [19] |
| *PROX1* | rs340874 | - | C | T | 1202/2520/1332 | 0.89 | 5054 | 0.92 | 0.49 | 0.49 | 0.5 | 0.55 | Prospero-related homeobox 1 | Fasting glucose [8] |
| *IGF1* | rs35767 | - | G | A | 3600/1333/145 | 0.12 | 5078 | 0.93 | 0.84 | 0.84 | 0.85 | 0.48 | Insulin-like growth factor 1 | Fasting insulin/T2D [8] |

| | | | | | | | | | | | | | | |
|---|---|---|---|---|---|---|---|---|---|---|---|---|---|---|
| *CYP2C19*2* | rs4244285 | - | A | G | 103/1132/3843 | 0.08 | 5078 | 0.93 | 0.13 | 0.14 | 0.13 | 0.10 | cytochrome P450, family 2, subfamily C, polypeptide 19 | Pharmacogenetic [5] |
| *IGF2BP2* | rs4402960 | - | T | G | 589/2138/2258 | 0.02 | 4985 | 0.91 | 0.33 | 0.34 | 0.33 | 0.37 | insulin-like growth factor 2 mRNA binding protein 2 | T2D [6] |
| *ZBED3* | rs4457053 | - | G | A | 258/1380/1755 | 0.58 | 3393 | 0.62 | 0.28 | 0.28 | 0.28 | 0.50 | zinc finger, BED-type containing 3 | T2D [11] |
| *ANKRD55* | rs459193 | - | G | A | 2626/2049/383 | 0.58 | 5058 | 0.93 | 0.72 | 0.72 | 0.72 | 0.70 | ankyrin repeat domain 55 | T2D [2] |
| *ADAMTS9* | rs4607103 | - | C | T | 2694/1978/373 | 0.72 | 5045 | 0.92 | 0.73 | 0.74 | 0.73 | 0.03 | ADAMTS9 antisense RNA 2 (non-protein coding) | T2D [9, 10] |
| *GCK* | rs4607517 | - | A | G | 80/1069/3911 | 0.47 | 5060 | 0.93 | 0.12 | 0.13 | 0.12 | 0.59 | Glucokinase | Fasting glucose/T2D [8] |
| *IRS1* | rs4675095 | - | A | T | 3131/310/15 | 0.03 | 3456 | 0.63 | 0.95 | 0.94 | 0.96 | 0.35 | insulin receptor substrate 1 | fasting glucose/HOMA-IR [8] |
| *CYP2C19*3* | rs4986893 | - | A | G | 0/13/5060 | 1.00 | 5073 | 0.93 | 0 | 0 | 0 | 0.81 | cytochrome P450, family 2, subfamily C, polypeptide 19 | Pharmacogenetic [5] |
| *ANK1* | rs516946 | - | C | T | 3048/1681/242 | 0.59 | 4971 | 0.91 | 0.78 | 0.78 | 0.79 | 0.85 | ankyrin 1, erythrocytic | T2D [2] |
| *KCNJ11* | rs5219 | - | T | C | 765/2296/1992 | 0.02 | 5053 | 0.92 | 0.38 | 0.38 | 0.38 | 0.73 | potassium inwardly-rectifying channel, subfamily J, member 11 | T2D [6] |
| *ABCB11* | rs560887 | - | C | T | 2448/2143/481 | 0.72 | 5072 | 0.93 | 0.69 | 0.7 | 0.69 | 0.69 | Glucose-6-phosphate, catalytic, 2 | Fasting glucose /T2D [8] |

| | | | | | | | | | | | | | | |
|---|---|---|---|---|---|---|---|---|---|---|---|---|---|---|
| *DUSP9* | rs5945326 | - | A | G | 2397/1135/267 | 0.00 | 4991 | 0.91 | 0.78 | 0.78 | 0.8 | 0.88 | dual specificity phosphatase 9 | T2D [11] |
| *INS* | rs689 | - | T | A | 2035/1497/273 | 0.93 | 3805 | 0.7 | 0.73 | 0.73 | 0.74 | 0.82 | Insulin gene | T1D/T2D [20] |
| *GRB10* | rs6943153 | - | T | C | 395/2058/2554 | 0.51 | 5007 | 0.92 | 0.28 | 0.26 | 0.29 | 0.04 | growth factor receptor-bound protein 10 | FG, FI [21] |
| *GLIS3* | rs7034200 | - | A | C | 1454/2467/1115 | 0.27 | 5036 | 0.92 | 0.53 | 0.52 | 0.54 | 0.74 | GLIS family zinc finger 3 | Fasting glucose /T2D [8] |
| *HMG20A* | rs7177055 | - | A | G | 2524/2070/456 | 0.29 | 5050 | 0.92 | 0.7 | 0.69 | 0.72 | 0.07 | high mobility group 20A | T2D [2] |
| *BCAR1* | rs7202877 | - | T | G | 3946/1048/84 | 0.14 | 5078 | 0.93 | 0.88 | 0.88 | 0.89 | 0.59 | breast cancer anti-estrogen resistance 1 | T2D [2] |
| *KIAA1486/IRS1* | rs7578326 | - | A | G | 2023/2293/699 | 0.24 | 5015 | 0.92 | 0.63 | 0.64 | 0.63 | 0.43 | insulin receptor substrate 1 | T2D [11] |
| *THADA* | rs7578597 | - | T | C | 4228/812/33 | 0.42 | 5073 | 0.93 | 0.91 | 0.91 | 0.92 | 0.76 | thyroid adenoma associated | T2D [9] |
| *RBMS* | rs7593730 | - | T | C | 140/1520/3411 | 0.06 | 5071 | 0.93 | 0.18 | 0.19 | 0.17 | 0.05 | RNA binding motif, single stranded interacting protein 1 | T2D [22] |
| *CDKAL1* | rs7754840 | - | C | G | 673/2295/2105 | 0.23 | 5073 | 0.93 | 0.36 | 0.36 | 0.36 | 0.57 | CDK5 regulatory subunit associated protein 1-like 1 | T2D [23] |
| *CDKAL1* | rs7756992 | - | G | A | 527/2131/2410 | 0.08 | 5068 | 0.93 | 0.31 | 0.32 | 0.32 | 0.31 | CDK5 regulatory subunit associated protein 1-like 1 | T2D [23] |
| *CYP3A5*3* | rs776746 | rs10211 | C | T | 2989/441/17 | 0.79 | 3447 | 0.63 | 0.93 | 0.94 | 0.92 | 0.62 | cytochrome P450, family 3, subfamily A, polypeptide 5 | Pharmacogenetic [24] |
| *TCF7L2* | rs7903146 | - | T | C | 383/2038/2633 | 0.70 | 5054 | 0.92 | 0.28 | 0.29 | 0.29 | 0.88 | transcription factor 7-like 2 (T-cell | T2D [25] |

| | | | | | | | | | | | | | | |
|---|---|---|---|---|---|---|---|---|---|---|---|---|---|---|
| | | | | | | | | | | | | | specific, HMG-box) | |
| *HNF1A* | rs7957197 | - | T | A | 3531/1366/167 | 0.02 | 5064 | 0.93 | 0.83 | 0.83 | 0.83 | 0.08 | HNF1 homeobox A | T2D [11] |
| *TSPAN8,LGR5* | rs7961581 | - | C | T | 364/1997/2690 | 0.83 | 5051 | 0.92 | 0.27 | 0.27 | 0.27 | 0.43 | tetraspanin 8 / leucine-rich repeat-containing G protein-coupled receptor 5 | T2D [9, 10] |
| *PRC1* | rs8042680 | - | A | C | 699/2334/2016 | 0.57 | 5049 | 0.92 | 0.37 | 0.37 | 0.37 | 0.74 | protein regulator of cytokinesis 1 | T2D [11] |
| *GIPR* | rs8108269 | - | G | T | 631/2060/1851 | 0.13 | 4542 | 0.83 | 0.37 | 0.36 | 0.36 | 0.96 | gastric inhibitory polypeptide receptor | T2D [2] |
| *JAZF1* | rs864745 | - | T | C | 1371/2548/1124 | 0.37 | 5043 | 0.92 | 0.52 | 0.53 | 0.53 | 0.10 | JAZF zinc finger 1 | T2D [9, 10] |
| *TP53INP1* | rs896854 | - | T | C | 1374/2513/1172 | 0.74 | 5059 | 0.93 | 0.52 | 0.52 | 0.52 | 0.89 | tumor protein p53 inducible nuclear protein 1 | T2D [11] |
| *FTO* | rs9939609 | - | A | T | 1061/2341/1385 | 0.23 | 4787 | 0.88 | 0.47 | 0.46 | 0.47 | 0.68 | Fat mass and obesity associated. | T2D/obesity [26] |

SNP=single-nucleotide polymorphism, RAF=risk allele frequency, HW P-value for Hardy-Weinberg equilibrium, Genotype counts (11/12/22) where 1 is risk allele, 2 is alternative allele, N – number of genotyped subjects, Call rate – percentage of genotyped participants ,P-value for chi-square test between famine exposed and unexposed groups to test if allele frequency differs significantly between the group due to famine exposure, Trait=original phenotype trait associated with the SNP.

| Supplementary Table 3. DNA variants and the risk of proliferative diabetic retinopathy in individuals perinatally exposed or unexposed to famine. | | | | | | | | | | | | | | | |
|---|---|---|---|---|---|---|---|---|---|---|---|---|---|---|---|
| Gene | SNP | Risk allele | RAF | N | Exposed to famine | | Unexposed | | Interaction | Heterogeneity | | HbA1c adjusted | | | |
| | | | | | | | | | | | | N | Interaction | Heterogeneity | |
| | | | | | N | OR (95% CI) | N | OR (95% CI) | P value | $I^2$ | Qep | | P value | $I^2$ | Qep |
| *WFS1* | rs10010131 | G | 0.62 | 3255 | 1606 | 0.78 (0.45 - 1.33) | 1649 | 0.91 (0.6 - 1.37) | 0.439 | 0 | 0.70 | 2300 | 0.77 | 0 | 0.86 |
| *SUGP1* | rs10401969 | C | 0.08 | 3239 | 1592 | 1.24 (0.47 - 3.29) | 1647 | 0.81 (0.39 - 1.68) | 0.718 | 0 | 0.57 | 2282 | 0.89 | 0 | 0.96 |
| *NAT2*6* | rs1041983 | C | 0.68 | 3252 | 1591 | 1.04 (0.58 - 1.85) | 1661 | 1.06 (0.69 - 1.64) | 0.912 | 0 | 0.96 | 2285 | 0.74 | 0 | 0.53 |
| *GIPR* | rs10423928 | A | 0.24 | 3262 | 1606 | 1.67 (0.93 - 2.99) | 1656 | 0.8 (0.5 - 1.27) | 0.148 | 62 | 0.10 | 2302 | 0.09 | 74 | 0.05 |
| *CYP2C8*3* | rs10509681 | C | 0.1 | 2300 | 1152 | 1.27 (0.43 - 3.72) | 1148 | 0.85 (0.37 - 1.97) | 0.715 | 0 | 0.63 | 1383 | 0.90 | 0 | 0.58 |
| *CDKN2B* | rs10811661 | T | 0.87 | 3242 | 1593 | 1.9 (0.75 - 4.8) | 1649 | 1.21 (0.63 - 2.32) | 0.256 | 0 | 0.51 | 2280 | 0.15 | 27 | 0.24 |
| *MTNR1B* | rs10830963 | G | 0.33 | 3289 | 1614 | 0.66 (0.38 - 1.15) | 1675 | 1.08 (0.72 - 1.61) | 0.219 | 27 | 0.24 | 2306 | 0.06 | 69 | 0.07 |
| *KLHDC5* | rs10842994 | C | 0.75 | 3267 | 1608 | 1.48 (0.81 - 2.7) | 1659 | 0.84 (0.54 - 1.29) | 0.286 | 37 | 0.21 | 2308 | 0.28 | 33 | 0.22 |
| *ADRA2A* | rs10885122 | G | 0.87 | 3280 | 1613 | 3.67 (1.77 - 7.63) | 1667 | 0.45 (0.28 - 0.71) | 0.003 | 94 | 0.00006* | 2299 | 0.04 | 90 | 0.003 |
| *NOTCH2* | rs10923931 | T | 0.11 | 3156 | 1543 | 1.1 (0.51 - 2.38) | 1613 | 0.81 (0.43 - 1.51) | 0.834 | 0 | 0.61 | 2226 | 0.61 | 0 | 0.52 |
| *CCND2* | rs11063069 | G | 0.24 | 3226 | 1578 | 1.16 (0.69 - 1.95) | 1648 | 0.96 (0.67 - 1.38) | 0.64 | 0 | 0.63 | 2282 | 0.91 | 0 | 0.89 |
| *FAM148B* | rs11071657 | A | 0.65 | 3267 | 1602 | 1.33 (0.79 - 2.23) | 1665 | 0.85 (0.59 - 1.22) | 0.362 | 27 | 0.24 | 2298 | 0.79 | 0 | 0.57 |
| *HHEX/IDE* | rs1111875 | C | 0.63 | 3267 | 1602 | 1.16 (0.71 - 1.91) | 1665 | 0.68 (0.47 - 0.97) | 0.616 | 53 | 0.15 | 2298 | 0.79 | 40 | 0.20 |
| *DCD* | rs1153188 | T | 0.25 | 3278 | 1609 | 2.18 (1.1 - 4.3) | 1669 | 0.44 (0.26 - 0.75) | 0.06 | 89 | 0.00 | 2305 | 0.03 | 89 | 0.00 |
| *CRY2* | rs11605924 | A | 0.5 | 3234 | 1598 | 0.72 (0.42 - 1.21) | 1636 | 1.3 (0.85 - 1.99) | 0.3 | 52 | 0.15 | 2275 | 0.41 | 12 | 0.29 |
| *ZFAND6* | rs11634397 | G | 0.66 | 3278 | 1608 | 0.71 (0.42 - 1.19) | 1670 | 0.97 (0.63 - 1.49) | 0.274 | 0 | 0.45 | 2306 | 0.63 | 0 | 0.97 |
| *ADCY5* | rs11708067 | A | 0.84 | 3229 | 1596 | 1.92 (1.01 - 3.64) | 1633 | 0.51 (0.33 - 0.79) | 0.095 | 87 | 0.01 | 2277 | 0.20 | 81 | 0.02 |
| *SLC2A2* | rs11920090 | T | 0.86 | 3238 | 1596 | 0.9 (0.43 - 1.88) | 1642 | 1.16 (0.67 - 1.98) | 0.818 | 0 | 0.66 | 2283 | 0.24 | 76 | 0.04 |
| *ZMIZ1* | rs12571751 | A | 0.52 | 3237 | 1593 | 1.32 (0.79 - 2.22) | 1644 | 0.81 (0.55 - 1.19) | 0.375 | 37 | 0.21 | 2279 | 0.19 | 45 | 0.18 |
| *MC4R* | rs12970134 | A | 0.24 | 3281 | 1611 | 1.33 (0.74 - 2.39) | 1670 | 1.04 (0.66 - 1.64) | 0.423 | 0 | 0.58 | 2308 | 0.68 | 0 | 0.82 |
| *SLC30A8* | rs13266634 | C | 0.73 | 3265 | 1601 | 0.95 (0.53 - 1.74) | 1664 | 1.04 (0.66 - 1.61) | 0.899 | 0 | 0.86 | 2297 | 0.82 | 0 | 0.85 |
| *TLE4* | rs13292136 | C | 0.96 | 3287 | 1612 | 0.37 (0.06 - 2.37) | 1675 | 3.28 (0.6 - 17.9) | 0.379 | 51 | 0.15 | - | - | - | - |
| *GRB14* | rs13389219 | C | 0.62 | 3260 | 1605 | 0.78 (0.47 - 1.32) | 1655 | 1.24 (0.83 - 1.86) | 0.44 | 25 | 0.25 | 2305 | 0.32 | 37 | 0.21 |

| | | | | | | | | | | | | | | | |
|---|---|---|---|---|---|---|---|---|---|---|---|---|---|---|---|
| *PLEKHH2* | rs1368086 | C | 0.8 | 2313 | 1161 | 1.72 (0.79 - 3.75) | 1152 | 0.97 (0.58 - 1.65) | 0.253 | 0 | 0.32 | 1386 | 0.45 | 52 | 0.15 |
| *HMGA2* | rs1531343 | C | 0.09 | 3281 | 1608 | 0.91 (0.38 - 2.14) | 1673 | 0.79 (0.4 - 1.54) | 0.85 | 0 | 0.83 | 2308 | 0.84 | 0 | 0.60 |
| *CENTD2* | rs1552224 | A | 0.86 | 3282 | 1611 | 0.78 (0.41 - 1.5) | 1671 | 0.96 (0.58 - 1.59) | 0.532 | 0 | 0.68 | 2308 | 0.64 | 0 | 0.69 |
| *KCNQ1* | rs163184 | G | 0.5 | 3471 | 1705 | 1.02 (0.62 - 1.68) | 1766 | 1.14 (0.75 - 1.71) | 0.94 | 0 | 0.79 | 2484 | 0.98 | 0 | 0.72 |
| *DGKB* | rs17168486 | T | 0.17 | 3256 | 1596 | 1.22 (0.68 - 2.18) | 1660 | 1.2 (0.79 - 1.83) | 0.581 | 0 | 0.98 | 2289 | 0.59 | 0 | 0.94 |
| *VPS13C* | rs17271305 | G | 0.37 | 3175 | 1558 | 1.02 (0.65 - 1.62) | 1617 | 1.15 (0.82 - 1.6) | 0.932 | 0 | 0.75 | 2287 | 0.13 | 29 | 0.24 |
| *FADS1* | rs174550 | T | 0.68 | 3282 | 1611 | 0.8 (0.46 - 1.42) | 1671 | 0.97 (0.61 - 1.53) | 0.53 | 0 | 0.68 | 2308 | 0.90 | 0 | 0.87 |
| *MC4R* | rs17782313 | C | 0.23 | 3087 | 1499 | 1.23 (0.68 - 2.23) | 1588 | 1.09 (0.69 - 1.74) | 0.566 | 0 | 0.79 | 2280 | 0.78 | 0 | 0.98 |
| *NAT2*5* | rs1799929 | C | 0.58 | 3280 | 1608 | 1.34 (0.81 - 2.2) | 1672 | 0.71 (0.47 - 1.06) | 0.335 | 63 | 0.10 | 2307 | 0.41 | 64 | 0.10 |
| *NAT2*6* | rs1799930 | A | 0.3 | 3220 | 1583 | 1.01 (0.58 - 1.77) | 1637 | 0.89 (0.59 - 1.32) | 0.967 | 0 | 0.75 | 2275 | 0.68 | 0 | 0.48 |
| *PPARG* | rs1801282 | C | 0.84 | 3425 | 1678 | 1.29 (0.63 - 2.66) | 1747 | 1.09 (0.65 - 1.84) | 0.555 | 0 | 0.75 | 2441 | 0.67 | 0 | 0.64 |
| *VEGF* | rs2010963 | G | 0.75 | 2009 | 1022 | 2.2 (1.06 - 4.58) | 987 | 0.7 (0.4 - 1.23) | 0.075 | 76 | 0.04 | 1147 | 0.18 | 55 | 0.14 |
| *TMEM195* | rs2191349 | T | 0.58 | 3261 | 1597 | 0.89 (0.53 - 1.5) | 1664 | 0.98 (0.66 - 1.46) | 0.723 | 0 | 0.82 | 2297 | 0.82 | 0 | 0.83 |
| *KCNQ1* | rs2237895 | A | 0.57 | 3429 | 1688 | 1.06 (0.65 - 1.71) | 1741 | 0.98 (0.67 - 1.43) | 0.849 | 0 | 0.83 | 2432 | 0.73 | 0 | 0.72 |
| *KCNQ1* | rs231362 | G | 0.53 | 3353 | 1637 | 0.7 (0.43 - 1.14) | 1716 | 1.14 (0.77 - 1.69) | 0.225 | 41 | 0.19 | 2379 | 0.62 | 0 | 0.52 |
| *BCL11A* | rs243021 | A | 0.47 | 3228 | 1589 | 0.7 (0.44 - 1.11) | 1639 | 1.15 (0.82 - 1.62) | 0.207 | 50 | 0.16 | 2275 | 0.03 | 88 | 0.00 |
| *BCL11A* | rs243088 | T | 0.54 | 3272 | 1603 | 1.05 (0.65 - 1.7) | 1669 | 1.08 (0.77 - 1.51) | 0.86 | 0 | 0.95 | 2291 | 0.38 | 33 | 0.22 |
| *PCSK9* | rs2479409 | G | 0.35 | 3268 | 1609 | 2.27 (1.26 - 4.06) | 1659 | 0.59 (0.37 - 0.94) | 0.021 | 89 | 0.00 | 2308 | 0.08 | 79 | 0.03 |
| *TLE1* | rs2796441 | G | 0.64 | 3267 | 1602 | 0.9 (0.53 - 1.53) | 1665 | 0.92 (0.59 - 1.43) | 0.742 | 0 | 0.95 | 2298 | 0.77 | 0 | 0.84 |
| *IRS1* | rs2943641 | C | 0.64 | 3288 | 1613 | 1.35 (0.81 - 2.24) | 1675 | 0.84 (0.57 - 1.24) | 0.335 | 31 | 0.23 | 2305 | 0.67 | 0 | 0.60 |
| *PROX1* | rs340874 | C | 0.49 | 3272 | 1607 | 0.54 (0.32 - 0.89) | 1665 | 1.57 (1.05 - 2.35) | 0.045 | 87 | 0.01 | 2291 | 0.03 | 87 | 0.01 |
| *IGF1* | rs35767 | G | 0.84 | 3282 | 1611 | 0.49 (0.24 - 0.99) | 1671 | 1.59 (0.91 - 2.76) | 0.096 | 79 | 0.03 | 2308 | 0.34 | 33 | 0.22 |
| *CYP2C19*2* | rs4244285 | A | 0.13 | 3281 | 1610 | 2.87 (1.23 - 6.68) | 1671 | 0.48 (0.23 - 0.99) | 0.04 | 86 | 0.01 | 2307 | 0.12 | 79 | 0.03 |
| *IGF2BP2* | rs4402960 | T | 0.33 | 3213 | 1573 | 1.58 (0.98 - 2.54) | 1640 | 0.98 (0.71 - 1.35) | 0.113 | 48 | 0.17 | 2282 | 0.54 | 0 | 0.69 |
| *ZBED3* | rs4457053 | G | 0.28 | 2003 | 1016 | 1.65 (0.86 - 3.2) | 987 | 0.87 (0.52 - 1.45) | 0.209 | 38 | 0.21 | 1150 | 0.08 | 78 | 0.03 |
| *ANKRD55* | rs459193 | G | 0.72 | 3267 | 1608 | 1.55 (0.88 - 2.73) | 1659 | 0.69 (0.46 - 1.03) | 0.199 | 73 | 0.05 | 2308 | 0.18 | 72 | 0.06 |
| *ADAMTS9* | rs4607103 | C | 0.73 | 3261 | 1600 | 0.86 (0.5 - 1.47) | 1661 | 0.69 (0.47 - 1.01) | 0.638 | 0 | 0.59 | 2296 | 0.67 | 56 | 0.13 |
| *GCK* | rs4607517 | A | 0.12 | 3275 | 1603 | 0.71 (0.32 - 1.59) | 1672 | 0.89 (0.49 - 1.6) | 0.48 | 0 | 0.71 | 2305 | 0.10 | 54 | 0.14 |
| *IRS1* | rs4675095 | A | 0.95 | 2045 | 1037 | 2.07 (0.57 - 7.51) | 1008 | 0.6 (0.22 - 1.67) | 0.351 | 35 | 0.22 | 1180 | 0.20 | 69 | 0.07 |

| | | | | | | | | | | | | | | | |
|---|---|---|---|---|---|---|---|---|---|---|---|---|---|---|---|
| *ANK1* | rs516946 | C | 0.78 | 3426 | 1678 | 1.05 (0.57 - 1.92) | 1748 | 1.23 (0.77 - 1.97) | 0.897 | 0 | 0.73 | 2443 | 0.39 | 0 | 0.62 |
| *KCNJ11* | rs5219 | T | 0.38 | 3264 | 1606 | 1.23 (0.74 - 2.03) | 1658 | 0.96 (0.63 - 1.44) | 0.506 | 0 | 0.53 | 2306 | 0.31 | 2 | 0.31 |
| *ABCB11* | rs560887 | C | 0.69 | 3280 | 1610 | 1.01 (0.6 - 1.68) | 1670 | 0.75 (0.5 - 1.11) | 0.982 | 0 | 0.45 | 2307 | 0.94 | 12 | 0.29 |
| *DUSP9* | rs5945326 | A | 0.78 | 3223 | 1583 | 0.95 (0.5 - 1.78) | 1640 | 1.44 (0.88 - 2.36) | 0.885 | 0 | 0.39 | 2272 | 0.86 | 0 | 0.39 |
| *INS* | rs689 | T | 0.73 | 3254 | 1588 | 0.9 (0.51 - 1.6) | 1666 | 1.07 (0.69 - 1.67) | 0.762 | 0 | 0.69 | 2277 | 0.71 | 0 | 0.43 |
| *GRB10* | rs6943153 | T | 0.28 | 3232 | 1589 | 0.69 (0.38 - 1.26) | 1643 | 1.07 (0.7 - 1.62) | 0.311 | 0 | 0.33 | 2278 | 0.19 | 70 | 0.07 |
| *GLIS3* | rs7034200 | A | 0.53 | 3259 | 1596 | 1.44 (0.84 - 2.46) | 1663 | 0.78 (0.51 - 1.21) | 0.269 | 51 | 0.15 | 2290 | 0.48 | 0 | 0.43 |
| *HMG20A* | rs7177055 | A | 0.7 | 3266 | 1601 | 0.79 (0.41 - 1.5) | 1665 | 1.9 (1.15 - 3.15) | 0.539 | 68 | 0.08 | 2298 | 0.49 | 69 | 0.07 |
| *BCAR1* | rs7202877 | T | 0.88 | 3281 | 1611 | 1.08 (0.5 - 2.33) | 1670 | 0.96 (0.54 - 1.7) | 0.876 | 0 | 0.84 | 2307 | 0.90 | 0 | 0.90 |
| *KIAA1486/IRS1* | rs7578326 | A | 0.63 | 3238 | 1595 | 1.07 (0.64 - 1.78) | 1643 | 1.09 (0.73 - 1.61) | 0.823 | 0 | 0.97 | 2281 | 0.66 | 0 | 0.71 |
| *THADA* | rs7578597 | T | 0.91 | 3288 | 1614 | 1.1 (0.39 - 3.08) | 1674 | 1.26 (0.59 - 2.7) | 0.885 | 0 | 0.85 | 2305 | 0.82 | 0 | 0.51 |
| *RBMS* | rs7593730 | T | 0.18 | 3281 | 1608 | 2.1 (0.96 - 4.62) | 1673 | 0.55 (0.28 - 1.1) | 0.121 | 77 | 0.04 | 2308 | 0.34 | 43 | 0.19 |
| *CDKAL1* | rs7754840 | C | 0.36 | 3287 | 1614 | 0.68 (0.42 - 1.1) | 1673 | 1.05 (0.75 - 1.47) | 0.188 | 31 | 0.23 | 2304 | 0.17 | 52 | 0.15 |
| *CDKAL1* | rs7756992 | G | 0.31 | 3283 | 1611 | 0.59 (0.35 - 1) | 1672 | 1.24 (0.87 - 1.77) | 0.1 | 73 | 0.05 | 2304 | 0.13 | 73 | 0.05 |
| *CYP3A5*3* | rs776746 | C | 0.93 | 3266 | 1601 | 3.03 (0.98 - 9.33) | 1665 | 0.97 (0.5 - 1.88) | 0.105 | 51 | 0.15 | 2298 | 0.48 | 0 | 0.67 |
| *TCF7L2* | rs7903146 | T | 0.28 | 3272 | 1602 | 1.03 (0.61 - 1.73) | 1670 | 1.55 (1.04 - 2.3) | 0.925 | 5 | 0.30 | 2295 | 0.80 | 46 | 0.18 |
| *HNF1A* | rs7957197 | T | 0.83 | 3273 | 1602 | 0.51 (0.24 - 1.08) | 1671 | 1.51 (0.85 - 2.69) | 0.141 | 72 | 0.06 | 2305 | 0.40 | 48 | 0.17 |
| *TSPAN8, LGR5* | rs7961581 | C | 0.27 | 3273 | 1606 | 0.55 (0.32 - 0.94) | 1667 | 1.57 (1.05 - 2.35) | 0.065 | 85 | 0.01 | 2296 | 0.11 | 76 | 0.04 |
| *PRC1* | rs8042680 | A | 0.37 | 3265 | 1602 | 0.75 (0.47 - 1.21) | 1663 | 1.01 (0.71 - 1.44) | 0.324 | 0 | 0.42 | 2296 | 0.14 | 44 | 0.18 |
| *GIPR* | rs8108269 | G | 0.37 | 2940 | 1461 | 1.73 (0.98 - 3.05) | 1479 | 0.75 (0.48 - 1.18) | 0.112 | 72 | 0.06 | 1985 | 0.05 | 85 | 0.01 |
| *JAZF1* | rs864745 | T | 0.52 | 3262 | 1599 | 0.91 (0.54 - 1.53) | 1663 | 1.25 (0.85 - 1.84) | 0.767 | 0 | 0.42 | 2295 | 0.50 | 0 | 0.72 |
| *TP53INP1* | rs896854 | T | 0.52 | 3268 | 1609 | 0.65 (0.4 - 1.03) | 1659 | 1.53 (1.1 - 2.13) | 0.126 | 84 | 0.01 | 2307 | 0.08 | 83 | 0.02 |
| *FTO* | rs9939609 | A | 0.47 | 3114 | 1518 | 0.65 (0.38 - 1.09) | 1596 | 1.43 (0.97 - 2.11) | 0.166 | 75 | 0.04 | 2240 | 0.13 | 79 | 0.03 |

All episodes of famine exposure were combined into decades of births before 1950 (exposed to famine) and after 1950 (unexposed). The odds ratios are obtained from interaction analyses between DNA variants (additive model) and combined famine exposure using a generalized estimation equation adjusted for sex, age at visit, and diabetes duration, and corrected for family relationships. The impact of DNA variants on the risk of diabetic retinopathy in individuals that had been born with or without famine exposure was also assessed using effect size heterogeneity Q-statistics and quantified using $I^2$ value [27]. RAF=risk allele frequency. Adj P-value= adjusted for sex, age at visit, diabetes duration, and corrected for family relationships, and with HbA1c as covariate.

*significant after adjustment for the multiple testing using Bonferroni correction ($p < 0.05$).

**Supplementary Table 4. Gene expression analyses in human retinal tissue.**

| Measurement | Diabetic donors | Non-diabetic donors | P-value |
|---|---|---|---|
| Number (M) | 5 (3) | 10 (6) | |
| Age (years), mean (DS) | 70 (8) | 67 (10) | n.s |
| Type of diabetes (1/2) | 0/5 | - | n.s |
| **Diabetes treatment** | | | |
| - oral agents | 2 | - | - |
| - insulin | 2 | - | - |
| - insulin + oral agents | 1 | - | - |
| **Cause of death** | | - | - |
| - Cardiovascular disease | 2 | 3 | - |
| - Cancer | 2 | 4 | - |
| - Others | 1 | 3 | - |
| Death to harvest (hours), mean (SD) | 3.4 (1.4) | 3.5 (1.6) | n.s |
| **Gene expression** | | | |
| *ADRA2A* | 6.9 (13.8) | 3.8 (6.1) | 0.56 |
| *PROX1* | 1.4 (0.4) | 1.7 (1.1) | 0.66 |

| Supplementary Table 5A. Association of DNA variants and famine exposure with the degree of methylation in the DOLCE study. | | | | | | | | | | |
|---|---|---|---|---|---|---|---|---|---|---|
| Gene | CpG | SNP | Risk allele | RAF | All participants | | | Exposed to famine *vs* unexposed | | |
| | | | | | N | Effect size (95% CI) | P-value | N | Effect size (95% CI) | P-value |
| *CYP2C19* | cg00051662 | rs4244285 | A | 0.13 | 78 | -0.19 (-0.31, -0.07) | 0.002* | 51 | 0.03 (-0.21, 0.27) | 0.824 |
| *PCSK9* | cg00815091 | rs2479409 | G | 0.35 | 78 | 0.00 (-0.06, 0.06) | 0.913 | 51 | 0.09 (-0.08, 0.26) | 0.301 |
| *PCSK9* | cg01189112 | rs2479409 | G | 0.35 | 78 | 0.01 (-0.06, 0.08) | 0.827 | 51 | -0.06 (-0.23, 0.11) | 0.514 |
| *ADRA2A* | cg02017450 | rs10885122 | G | 0.86 | 80 | -0.01 (-0.16, 0.14) | 0.914 | 51 | 0.34 (0.05, 0.63) | 0.027 |
| *PROX1* | cg03957440 | rs340874 | C | 0.5 | 80 | -0.08 (-0.16, 0.00) | 0.047 | 51 | -0.03 (-0.26, 0.20) | 0.794 |
| *PCSK9* | cg06197377 | rs2479409 | G | 0.35 | 78 | 0.03 (-0.06, 0.11) | 0.529 | 51 | -0.04 (-0.19, 0.10) | 0.579 |
| *ADRA2A* | cg09009466 | rs10885122 | G | 0.86 | 80 | 0.03 (-0.07, 0.13) | 0.524 | 51 | 0.05 (-0.10, 0.20) | 0.534 |
| *PCSK9* | cg13191808 | rs2479409 | G | 0.35 | 78 | 0.04 (-0.02, 0.10) | 0.192 | 51 | -0.06 (-0.25, 0.12) | 0.521 |
| *PCSK9* | cg14089067 | rs2479409 | G | 0.35 | 78 | 0.00 (-0.08, 0.09) | 0.919 | 51 | -0.05 (-0.22, 0.13) | 0.608 |
| *PCSK9* | cg14615807 | rs2479409 | G | 0.35 | 78 | -0.03 (-0.09, 0.04) | 0.430 | 51 | 0.01 (-0.12, 0.15) | 0.834 |
| *PCSK9* | cg17167852 | rs2479409 | G | 0.35 | 78 | 0.04 (-0.07, 0.15) | 0.474 | 51 | 0.01 (-0.20, 0.23) | 0.907 |
| *PROX1* | cg17954152 | rs340874 | C | 0.50 | 80 | 0.04 (-0.06, 0.15) | 0.434 | 51 | -0.12 (-0.36, 0.11) | 0.318 |
| *PCSK9* | cg19971655 | rs2479409 | G | 0.35 | 78 | -0.02 (-0.08, 0.04) | 0.451 | 51 | 0.01 (-0.12, 0.13) | 0.931 |
| *PROX1* | cg22176895 | rs340874 | C | 0.50 | 80 | 0.09 (0.02, 0.16) | 0.010 | 51 | 0.01 (-0.15, 0.16) | 0.930 |
| *PROX1* | cg23084895 | rs340874 | C | 0.50 | 80 | 0.07 (0.01, 0.14) | 0.033 | 51 | -0.09 (-0.23, 0.06) | 0.242 |
| *PROX1* | cg23260547 | rs340874 | C | 0.50 | 80 | 0.07 (-0.04, 0.18) | 0.204 | 51 | 0.23 (-0.01, 0.47) | 0.069 |
| *PROX1* | cg24083324 | rs340874 | C | 0.50 | 80 | 0.04 (-0.05, 0.13) | 0.344 | 51 | 0.01 (-0.17, 0.20) | 0.895 |
| *PCSK9* | cg26666107 | rs2479409 | G | 0.35 | 78 | 0.01 (-0.06, 0.08) | 0.794 | 51 | -0.04 (-0.20, 0.13) | 0.648 |
| *ADRA2A* | cg26691305 | rs10885122 | G | 0.86 | 80 | 0.03 (-0.06, 0.13) | 0.496 | 51 | -0.02 (-0.22, 0.19) | 0.875 |

Association of DNA variants and famine exposure (exposed, *n*=43, *vs.* unexposed, *n*=8) with the degree of methylation was analyses using linear regression adjusted for sex and age at visit. RAF=risk allele frequency.

*significant after adjustment for the multiple testing using Bonferroni correction (p-value< 0.05).

| Supplementary Table 5B. Differentially methylated CpG sites in patients with type 2 diabetes and non-diabetic participants of the DOLCE study | | | | | | | | | | | | | | | | | | | | | | |
|---|---|---|---|---|---|---|---|---|---|---|---|---|---|---|---|---|---|---|---|---|---|---|
| **CpG** | **LogFC** | **AveExpr** | **t** | **P-value** | **Adj. P-value** | **B** | **T_AVG** | **C_AVG** | **Delta Beta** | **CHR** | **MAPINFO** | **Strand** | **Type** | **Gene** | **Feature** | **Cgi** | **Feat.cgi** | **UCSC CpG islands name** | **DHS** | **Enhancer** | **Phantom** | **Probe SNPs** |
| ***PCSK9*** | | | | | | | | | | | | | | | | | | | | | | |
| cg06197377 | 0.024 | 0.349 | 2.948 | 0.004 | 0.048 | -4.026 | 0.366 | 0.342 | -0.024 | 1 | 55505061 | R | II | PCSK9 | TSS200 | island | TSS200-island | chr1:55505060-55506015 | TRUE | TRUE | | |
| cg26666107 | 0.015 | 0.142 | 3.042 | 0.003 | 0.04 | -3.771 | 0.152 | 0.138 | -0.015 | 1 | 55505073 | R | II | PCSK9 | TSS200 | island | TSS200-island | chr1:55505060-55506015 | TRUE | TRUE | | |
| cg17167852 | 0.017 | 0.083 | 3.567 | 0.001 | 0.014 | -2.226 | 0.095 | 0.078 | -0.017 | 1 | 55505189 | F | I | PCSK9 | TSS200 | island | TSS200-island | chr1:55505060-55506015 | TRUE | TRUE | high-CpG:55277777-55277829 | rs72658888 |
| cg13191808 | 0.008 | 0.07 | 3.078 | 0.003 | 0.037 | -3.671 | 0.076 | 0.067 | -0.008 | 1 | 55505327 | R | II | PCSK9 | 1stExon | island | 1stExon-island | chr1:55505060-55506015 | NA | TRUE | | |
| | | | | | | | | | | | | | | | | | | | | | | |
| ***CYP2C19*** | | | | | | | | | | | | | | | | | | | | | | |
| cg00051662 | -0.018 | 0.878 | -3.41 | 0.001 | 0.019 | -2.709 | 0.866 | 0.884 | 0.018 | 10 | 96521086 | F | II | CYP2C19 | TSS1500 | opensea | TSS1500-opensea | | NA | NA | | |
| | | | | | | | | | | | | | | | | | | | | | | |
| ***ADRA2A*** | | | | | | | | | | | | | | | | | | | | | | |
| cg02017450 | -0.008 | 0.044 | -3.088 | 0.003 | 0.036 | -3.644 | 0.039 | 0.046 | 0.008 | 10 | 10631670 | F | II | | IGR | opensea | IGR-opensea | | NA | TRUE | | |
| cg09009466 | -0.024 | 0.759 | -3.7 | 0 | 0.011 | -1.805 | 0.743 | 0.767 | 0.024 | 10 | 11483241 | R | I | | IGR | opensea | IGR-opensea | | NA | NA | | |
| | | | | | | | | | | | | | | | | | | | | | | |
| ***PROX1*** | | | | | | | | | | | | | | | | | | | | | | |
| cg10288510 | 0.023 | 0.17 | 3.305 | 0.001 | 0.024 | -3.02 | 0.186 | 0.163 | -0.023 | 1 | 214158727 | R | II | | IGR | island | IGR-island | chr1:214158726-214159080 | NA | TRUE | | |
| cg17954152 | 0.03 | 0.19 | 3.372 | 0.001 | 0.021 | -2.822 | 0.211 | 0.181 | -0.03 | 1 | 214160860 | F | II | PROX1 | TSS1500 | island | TSS1500-island | chr1:214160798-214161034 | NA | NA | | rs78824623 |
| cg23260547 | 0.012 | 0.053 | 3.995 | 0 | 0.006 | -0.837 | 0.061 | 0.049 | -0.012 | 1 | 214161371 | R | I | PROX1 | TSS1500 | island | TSS1500-island | chr1:214161197-214161415 | NA | NA | | |

| | | | | | | | | | | | | | | | | | | | | | | |
|---|---|---|---|---|---|---|---|---|---|---|---|---|---|---|---|---|---|---|---|---|---|---|
| cg22176895 | 0.014 | 0.125 | 3.61 | 0.001 | 0.013 | -2.09 | 0.134 | 0.121 | -0.014 | 1 | 214162039 | R | II | PROX1 | 5'UTR | shore | 5'UTR-shore | chr1:214162508-214162737 | NA | NA | | rs76992635 |
| cg24083324 | 0.007 | 0.049 | 3.053 | 0.003 | 0.039 | -3.739 | 0.053 | 0.047 | -0.007 | 1 | 214162604 | R | I | PROX1 | 5'UTR | island | 5'UTR-island | chr1:214162508-214162737 | NA | NA | | |
| cg23084895 | 0.01 | 0.09 | 3.267 | 0.002 | 0.026 | -3.131 | 0.097 | 0.087 | -0.01 | 1 | 214162854 | R | II | PROX1 | 5'UTR | shore | 5'UTR-shore | chr1:214160798-214161034 | NA | NA | | |
| cg03957440 | -0.043 | 0.572 | -3.46 | 0.001 | 0.018 | -2.555 | 0.542 | 0.585 | 0.043 | 1 | 214209324 | R | II | PROX1 | 3'UTR | opensea | 3'UTR-opensea | | NA | NA | | |
| cg07785556 | -0.018 | 0.856 | -3.669 | 0 | 0.012 | -1.904 | 0.843 | 0.861 | 0.018 | 1 | 214358088 | R | II | | IGR | shelf | IGR-shelf | chr1:214360607-214360965 | NA | NA | | |

CpGs showing significant differences in the degree of methylation between individuals with type 2 diabetes and control individuals (diabetic, *n*=53, non-diabetic individuals, *n*=28) for the selected genetic loci associated with diabetic retinopathy after exposure to famine. logFC=log fold-change; *t*=test statistic; adj.P-value=*P*-values adjusted for multiple testing correction using false discovery rate method; delta beta=difference in beta values between cases and controls; CHR, chromosome.

**Supplementary Table 6. Association of DNA variants with the degree of methylation in the Avon Longitudinal Study of Parents and Children cohort.**

| Gene | SNP | Risk allele (DOLCE) | RAF (DOLCE) | Timepoint | Chr | Position | A1 (RISK) | A2 (REFERENCE) | MAF | CpG | CpG Chr | CpG Position | Beta | T-stat | Effect size | P-value | Trans |
|---|---|---|---|---|---|---|---|---|---|---|---|---|---|---|---|---|---|
| *ADRA2A* | rs10885122 | G | 0.86 | Middle Age | 10 | 113042093 | T | G | 0.13 | cg19515510 | 17 | 73201236 | 0.27 | 5.44 | 0.00021 | 0 | Y |
| *PCSK9* | rs2479409 | G | 0.35 | Childhood | 1 | 55504650 | G | A | 0.30 | cg02091786 | 11 | 62415525 | 0.24 | 5.58 | 0.00972 | 0 | Y |
| *PCSK9* | rs2479409 | G | 0.35 | Childhood | 1 | 55504650 | G | A | 0.30 | cg10476584 | 17 | 77788722 | -0.25 | -5.56 | 0.00087 | 0 | Y |
| *PCSK9* | rs2479409 | G | 0.35 | Middle Age | 1 | 55504650 | G | A | 0.32 | cg09902229 | 20 | 57582292 | -0.26 | -5.59 | 0.00155 | 0 | Y |
| *PCSK9* | rs2479409 | G | 0.35 | Childhood | 1 | 55504650 | G | A | 0.30 | cg17826594 | 1 | 55506014 | -0.25 | -5.73 | 0.00102 | 0 | N |
| *PCSK9* | rs2479409 | G | 0.35 | Childhood | 1 | 55504650 | G | A | 0.30 | cg01519238 | 8 | 145515711 | -0.23 | -5.48 | 0.00069 | 0 | Y |
| *PCSK9* | rs2479409 | G | 0.35 | Middle Age | 1 | 55504650 | G | A | 0.32 | cg19215386 | 18 | 72921446 | -0.22 | -5.81 | 0.00179 | 0 | Y |
| *PROX1* | rs340874 | C | 0.50 | Birth | 1 | 214159256 | T | C | 0.43 | cg20692998 | 1 | 214159203 | -0.26 | -5.77 | 0.00324 | 0 | N |
| *PROX1* | rs340874 | C | 0.50 | Pregnancy | 1 | 214159256 | T | C | 0.43 | cg26293546 | 1 | 214162659 | -0.24 | -5.81 | 0.0028 | 0 | N |
| *PROX1* | rs340874 | C | 0.50 | Adolescence | 1 | 214159256 | T | C | 0.42 | cg10288510 | 1 | 214158727 | -0.31 | -8.12 | 0.00251 | 0 | N |
| *PROX1* | rs340874 | C | 0.50 | Childhood | 1 | 214159256 | T | C | 0.43 | cg10288510 | 1 | 214158727 | -0.3 | -8.11 | 0.0033 | 0 | N |
| *PROX1* | rs340874 | C | 0.50 | Middle Age | 1 | 214159256 | T | C | 0.43 | cg10288510 | 1 | 214158727 | -0.33 | -7.15 | 0.00313 | 0 | N |
| *PROX1* | rs340874 | C | 0.50 | Adolescence | 1 | 214159256 | T | C | 0.42 | cg26293546 | 1 | 214162659 | -0.21 | -5.85 | 0.00314 | 0 | N |
| *PROX1* | rs340874 | C | 0.50 | Adolescence | 1 | 214159256 | T | C | 0.42 | cg18318878 | 1 | 214159037 | -0.24 | -5.46 | 0.00444 | 0 | N |
| *PROX1* | rs340874 | C | 0.50 | Middle Age | 1 | 214159256 | T | C | 0.43 | cg20692998 | 1 | 214159203 | -0.28 | -5.95 | 0.00532 | 0 | N |
| *PROX1* | rs340874 | C | 0.50 | Adolescence | 1 | 214159256 | T | C | 0.42 | cg20692998 | 1 | 214159203 | -0.34 | -8.14 | 0.00529 | 0 | N |
| *PROX1* | rs340874 | C | 0.50 | Pregnancy | 1 | 214159256 | T | C | 0.43 | cg10288510 | 1 | 214158727 | -0.24 | -5.64 | 0.00219 | 0 | N |
| *PROX1* | rs340874 | C | 0.50 | Middle Age | 1 | 214159256 | T | C | 0.43 | cg26293546 | 1 | 214162659 | -0.26 | -6.14 | 0.00213 | 0 | N |
| *PROX1* | rs340874 | C | 0.50 | Pregnancy | 1 | 214159256 | T | C | 0.43 | cg20692998 | 1 | 214159203 | -0.35 | -7.77 | 0.00577 | 0 | N |
| *PROX1* | rs340874 | C | 0.50 | Childhood | 1 | 214159256 | T | C | 0.43 | cg20692998 | 1 | 214159203 | -0.39 | -9.73 | 0.00533 | 0 | N |
| *CYP2C19*2* | rs4244285 | A | 0.13 | Adolescence | 10 | 96541616 | A | G | 0.15 | cg20031717 | 10 | 96523248 | 0.49 | 8.77 | 0.04824 | 0 | N |
| *CYP2C19*2* | rs4244285 | A | 0.13 | Childhood | 10 | 96541616 | A | G | 0.15 | cg20031717 | 10 | 96523248 | 0.48 | 8.46 | 0.03882 | 0 | N |

| | | | | | | | | | | | | | | | | | |
|---|---|---|---|---|---|---|---|---|---|---|---|---|---|---|---|---|---|
| *CYP2C19*2* | rs4244285 | A | 0.13 | Childhood | 10 | 96541616 | A | G | 0.15 | cg04189838 | 10 | 96523347 | 0.58 | 10.21 | 0.02094 | 0 | N |
| *CYP2C19*2* | rs4244285 | A | 0.13 | Pregnancy | 10 | 96541616 | A | G | 0.15 | cg20031717 | 10 | 96523248 | 0.37 | 6.22 | 0.02853 | 0 | N |
| *CYP2C19*2* | rs4244285 | A | 0.13 | Adolescence | 10 | 96541616 | A | G | 0.15 | cg04757345 | 4 | 128918835 | -0.32 | -5.73 | 0.035 | 0 | Y |
| *CYP2C19*2* | rs4244285 | A | 0.13 | Adolescence | 10 | 96541616 | A | G | 0.15 | cg00051662 | 10 | 96521086 | -0.31 | -5.82 | 0.02447 | 0 | N |
| *CYP2C19*2* | rs4244285 | A | 0.13 | Middle Age | 10 | 96541616 | A | G | 0.15 | cg04189838 | 10 | 96523347 | 0.45 | 7.33 | 0.01493 | 0 | N |
| *CYP2C19*2* | rs4244285 | A | 0.13 | Adolescence | 10 | 96541616 | A | G | 0.15 | cg04189838 | 10 | 96523347 | 0.52 | 9.16 | 0.02695 | 0 | N |
| *CYP2C19*2* | rs4244285 | A | 0.13 | Childhood | 10 | 96541616 | A | G | 0.15 | cg00051662 | 10 | 96521086 | -0.35 | -6.71 | 0.00775 | 0 | N |
| *CYP2C19*2* | rs4244285 | A | 0.13 | Pregnancy | 10 | 96541616 | A | G | 0.15 | cg04189838 | 10 | 96523347 | 0.57 | 9.32 | 0.02373 | 0 | N |

Analyses were performed using dataset MatrixEqtl (downloaded from http://www.mqtldb.org/search.htm). [28]

| **Supplementary Table 7. Association of DNA variants with gene expression in the GTEX database.** | | | | | | | | | | | |
|---|---|---|---|---|---|---|---|---|---|---|---|
| **Gene** | **SNP** | **Risk allele** | **RAF** | **Chr** | **BP** | **Reference allele** | **Alternative allele** | **MAF** | **P-value** | **Effect_size** | **Tissue** |
| *PCSK9* | rs2479409 | G | 0.35 | 1 | 55504650 | G | A | 0.33 | <0.001 | 0.20 | Skin_Sun_Exposed_Lower_leg |
| *PROX1* | rs340874 | C | 0.50 | 1 | 214159256 | T | C | 0.45 | <0.001 | 0.32 | Brain_Cerebellum |
| *PROX1* | rs340874 | C | 0.50 | 1 | 214159256 | T | C | 0.46 | <0.001 | 0.32 | Brain_Cerebellar_Hemisphere |
| *PROX1* | rs340874 | C | 0.50 | 1 | 214159256 | T | C | 0.48 | <0.001 | 0.25 | Brain_Putamen_basal_ganglia |
| *CYP2C19*2* | rs4244285 | A | 0.13 | 10 | 96541616 | G | A | 0.14 | <0.001 | 0.55 | Skin_Sun_Exposed_Lower_leg |
| *CYP2C19*2* | rs4244285 | A | 0.13 | 10 | 96541616 | G | A | 0.13 | <0.001 | 0.57 | Esophagus_Mucosa |
| *CYP2C19*2* | rs4244285 | A | 0.13 | 10 | 96541616 | G | A | 0.13 | <0.001 | 0.44 | Esophagus_Mucosa |
| *CYP2C19*2* | rs4244285 | A | 0.13 | 10 | 96541616 | G | A | 0.14 | <0.001 | 0.32 | Stomach |
| *CYP2C19*2* | rs4244285 | A | 0.13 | 10 | 96541616 | G | A | 0.14 | <0.001 | 0.16 | Skin_Sun_Exposed_Lower_leg |
| *CYP2C19*2* | rs4244285 | A | 0.13 | 10 | 96541616 | G | A | 0.12 | <0.001 | 0.45 | Liver |
| *CYP2C19*2* | rs4244285 | A | 0.13 | 10 | 96541616 | G | A | 0.13 | <0.001 | 0.69 | Esophagus_Mucosa |
| *CYP2C19*2* | rs4244285 | A | 0.13 | 10 | 96541616 | G | A | 0.14 | <0.001 | 0.51 | Skin_Not_Sun_Exposed_Suprapubic |

Analysis was performed using dataset GTEx_Analysis_v7_eQTL.tar.gz database (downloaded from https://gtexportal.org/home/datasets).
The genotypes of ADRA2A or its proxies were not present in the GTEX database, and therefore these data were not available for the analyses.

**References**


1. Sandhu, M.S., et al., *Common variants in WFS1 confer risk of type 2 diabetes.* Nature genetics, 2007. **39**(8): p. 951.
2. Morris, A.P., et al., *Large-scale association analysis provides insights into the genetic architecture and pathophysiology of type 2 diabetes.* Nature genetics, 2012. **44**(9): p. 981.
3. Vatsis, K.P., K.J. Martell, and W.W. Weber, *Diverse point mutations in the human gene for polymorphic N-acetyltransferase.* Proceedings of the National Academy of Sciences, 1991. **88**(14): p. 6333-6337.
4. Saxena, R., et al., *Genetic variation in GIPR influences the glucose and insulin responses to an oral glucose challenge.* Nature genetics, 2010. **42**(2): p. 142.
5. Miners, J.O. and D.J. Birkett, *Cytochrome P4502C9: an enzyme of major importance in human drug metabolism.* British journal of clinical pharmacology, 1998. **45**(6): p. 525-538.
6. Saxena, R., et al., *Genome-wide association analysis identifies loci for type 2 diabetes and triglyceride levels.* Science, 2007. **316**(5829): p. 1331-1336.
7. Prokopenko, I., et al., *Variants in MTNR1B influence fasting glucose levels.* Nature genetics, 2009. **41**(1): p. 77.
8. Dupuis, J., et al., *New genetic loci implicated in fasting glucose homeostasis and their impact on type 2 diabetes risk.* Nature genetics, 2010. **42**(2): p. 105.
9. Zeggini, E., et al., *Meta-analysis of genome-wide association data and large-scale replication identifies additional susceptibility loci for type 2 diabetes.* Nat Genet, 2008. **40**(5): p. 638-45.
10. Lyssenko, V., et al., *Clinical risk factors, DNA variants, and the development of type 2 diabetes.* New England Journal of Medicine, 2008. **359**(21): p. 2220-2232.
11. Voight, B.F., et al., *Twelve type 2 diabetes susceptibility loci identified through large-scale association analysis.* Nature genetics, 2010. **42**(7): p. 579.
12. Chambers, J.C., et al., *Common genetic variation near MC4R is associated with waist circumference and insulin resistance.* Nature genetics, 2008. **40**(6): p. 716.
13. Sladek, R., et al., *A genome-wide association study identifies novel risk loci for type 2 diabetes.* Nature, 2007. **445**(7130): p. 881.
14. Yasuda, K., et al., *Variants in KCNQ1 are associated with susceptibility to type 2 diabetes mellitus.* Nature genetics, 2008. **40**(9): p. 1092.
15. Willer, C.J., et al., *Six new loci associated with body mass index highlight a neuronal influence on body weight regulation.* Nature genetics, 2009. **41**(1): p. 25.
16. Thorleifsson, G., et al., *Genome-wide association yields new sequence variants at seven loci that associate with measures of obesity.* Nature genetics, 2009. **41**(1): p. 18.
17. Ng, D.P., *Human genetics of diabetic retinopathy: current perspectives.* Journal of ophthalmology, 2010. **2010**.
18. Teslovich, T.M., et al., *Biological, clinical and population relevance of 95 loci for blood lipids.* Nature, 2010. **466**(7307): p. 707.
19. Rung, J., et al., *Genetic variant near IRS1 is associated with type 2 diabetes, insulin resistance and hyperinsulinemia.* Nature genetics, 2009. **41**(10): p. 1110.
20. Cervin, C., et al., *Genetic similarities between latent autoimmune diabetes in adults, type 1 diabetes, and type 2 diabetes.* Diabetes, 2008. **57**(5): p. 1433-1437.
21. Barroso, I. and R. Scott, *Genome-Wide Association Studies of Quantitative Glycaemic Traits*, in *The Genetics of Type 2 Diabetes and Related Traits*. 2016, Springer. p. 63-89.

22. Qi, L., et al., *Genetic variants at 2q24 are associated with susceptibility to type 2 diabetes.* Human molecular genetics, 2010. **19**(13): p. 2706-2715.
23. Steinthorsdottir, V., et al., *A variant in CDKAL1 influences insulin response and risk of type 2 diabetes.* Nature genetics, 2007. **39**(6): p. 770.
24. Willrich, M.A.V., et al., *Effects of atorvastatin on CYP3A4 and CYP3A5 mRNA expression in mononuclear cells and CYP3A activity in hypercholeresterolemic patients.* Clinica Chimica Acta, 2013. **421**: p. 157-163.
25. Grant, S.F., et al., *Variant of transcription factor 7-like 2 (TCF7L2) gene confers risk of type 2 diabetes.* Nature genetics, 2006. **38**(3): p. 320.
26. Consortium, W.T.C.C., *Genome-wide association study of 14,000 cases of seven common diseases and 3,000 shared controls.* Nature, 2007. **447**(7145): p. 661.
27. Fleiss, J.L., B. Levin, and M.C. Paik, *Statistical methods for rates and proportions*. 2013: John Wiley & Sons.
28. Gaunt, T.R., et al., *Systematic identification of genetic influences on methylation across the human life course.* Genome biology, 2016. **17**(1): p. 61.
